# Synthesis and Characterization of Novel Cobalt Carbonyl Phosphorus and Arsenic Clusters

**DOI:** 10.3390/molecules29092025

**Published:** 2024-04-27

**Authors:** Mehdi Elsayed Moussa, Susanne Bauer, Christian Graßl, Christoph Riesinger, Gábor Balázs, Manfred Scheer

**Affiliations:** Department of Inorganic Chemistry, University of Regensburg, 93040 Regensburg, Germany; mehdi.elsayed-mousa@chemie.uni-regensburg.de (M.E.M.); susanne.bauer@chemie.uni-regensburg.de (S.B.); christian.grassl@chemie.uni-regensburg.de (C.G.); christoph.riesinger@chemie.uni-regensburg.de (C.R.); gabor.balazs@chemie.uni-regensburg.de (G.B.)

**Keywords:** phosphorus, arsenic, cobalt clusters, carbonyl, interstitial

## Abstract

Phosphorus- and arsenic-containing cobalt clusters are an interesting class of compounds that continue to provide new structures with captivating bonding patterns. Although the first members of this family were reported 45 years ago, the number of such species is still limited within the broad family of transition metal complexes bearing pnictogen atoms. Herein, we present the reaction of Co_2_(CO)_8_ as a cobalt source with a number of phosphorus- and arsenic-containing compounds under variable reaction conditions. These reactions result in various known and novel cobalt phosphorus and cobalt arsenic clusters in which different nuclearity ratios between P/As and Co exist. All those clusters were characterized by X-ray structural analysis and partly by IR, ^31^P{^1^H} NMR, EI-MS and elemental analysis. This comprehensive study is the first detailed study in this field that reveals the richness of compounds that could be obtained only by modifying the ratio of used reactants and the involved reaction conditions.

## 1. Introduction

Transition metal complexes that incorporate group 15 elements have attracted increasing interest in the past three decades mainly due to their unprecedented structures [1] and flexible coordination behaviors, which render them versatile building blocks in supramolecular chemistry [1,2,3,4]. Within this field, cobalt clusters containing phosphorus and arsenic atoms have emerged as active materials for catalysis [5,6,7], magnetism [8], and as potential precursors for CoP nanoparticles [9]. In 1969, the Dahl group reported the first examples in this field, which included the tetrahedral arsenic-cobalt carbonyl clusters As_3_{Co(CO)_3_} and As_2_{Co(CO)_3_}_2_ obtained from the reaction of Co_2_(CO)_8_ with [AsCH_3_]_5_ and AsCl_3_, respectively [10,11]. Later on, the complex [µ_4_-AsCo_3_(CO)_9_]_3_ [12] was also isolated, which demonstrated the interchangeable roles of As and Co(CO)_3_ units. Meanwhile, Markó et al. isolated the analogous phosphorus derivative Co_2_(CO)_6_P_2_ from the reaction of Na[Co(CO)_4_] with PX_3_ (X = Cl, Br) [13]. Additionally, the tetrahedral compounds P_3_Co(CO)_3_ and PCo_3_(CO)_9_ were isolated by the Orosz group from the reaction of Co_2_(CO)_8_ with white phosphorus (P_4_) and PI_3_, respectively [14]. The groups of Seyferth and Nixon isolated the tetrahedral phosphorus cobalt clusters [(RCP)Co_2_(CO)_6_)] (R = CH_3_, *t*Bu, Ph, SiMe_3_) from the reaction of Co_2_(CO)_8_ with RCCl_2_PCl_2_ [15] and *t*BuCP [16]. More recently, a number of more complex anionic P- [8,17] and As-containing cobalt clusters [18,19] with higher Co nuclearities were synthesized from the reaction of Na[Co(CO)_4_] with variable P- and As-starting materials. Our group contributed to this field by developing new strategies for the synthesis of cobalt clusters incorporating pnictogen atoms. In one approach, the formation of large P_n_ species was attainable from the reaction of P_4_ with the triple-decker complex [(Cp′′′Co)_2_(*η*^4^:*η*^4^-C_7_H_8_)] (Cp′′′ = 1,2,4-C_5_H_2_(*t*Bu)_3_), which dissociates in solution to give 14 VE (valence electron) Cp′′′Co moieties [20]. In that respect, a controlled synthesis of P_4_, P_8_, P_12_, P_16_ and even P_24_-containing cobalt complexes proved achievable. This approach was more recently extended to the reaction with As_4_ allowing the synthesis of As_4_, As_10_ and As_12_ cobalt clusters [21]. In another approach, the sandwich complex [Cp′′′Co(*η*^4^-P_4_)] was found to dimerize in solution forming P_8_-containing cobalt complexes [22]. In addition to the aforementioned compounds, only a few other clusters of this type are known and are thus still relatively limited within the broad family of transition metal compounds containing phosphorus atoms [1,23]. Therefore, further investigations to enrich the library of this family of compounds with new candidates, as well as to understand reaction conditions that allow us to obtain them selectively, are still of current interest. Herein, we present the reaction of Co(CO)_8_ with the phosphorus and arsenic sources: “[Cr(CO)_4+n_(PH_3_)_2−n_] (*n* = 0, 1), [Fe(CO)_4_(PH_3_)], P(SiMe_3_)_3_, P_4_, As_4_, [W(CO)_5_(AsH_3_)] and As(SiMe_3_)_3_”. These reactions afforded a variety of known as well as novel cobalt carbonyl clusters incorporating P and As atoms. Interestingly, most of the known compounds are obtained in much better yields via our novel synthetic strategies presented herein, thus allowing the completion of their characterizations, including X-ray structure analysis.

## 2. Results and Discussion

### 2.1. Synthesis and X-ray Structures of Compound ***1*** and the Cobalt Clusters [{M(CO)_n_}{Co(CO)_3_}_3_E] (n = 5, M = Cr, E = P (***2***), M = W, E = As (***3***); n = 4, M = Fe, E = P (***4***))

**Scheme 1:**
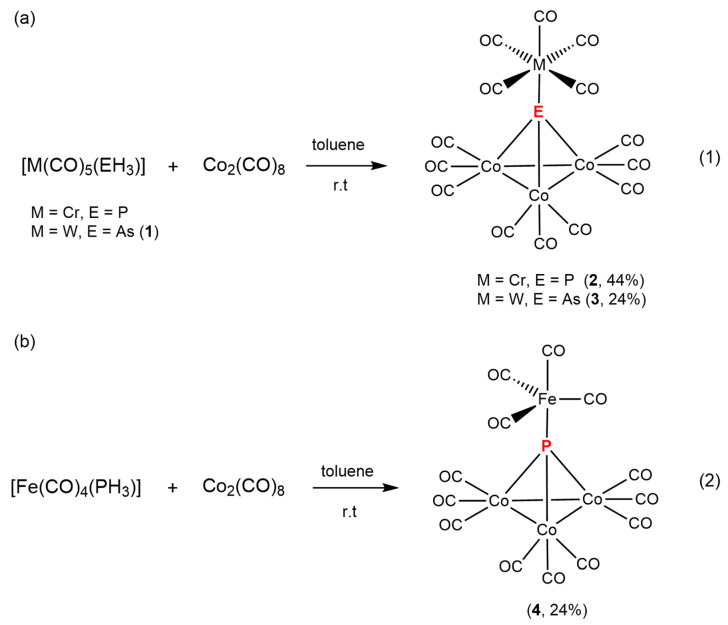
Synthesis of the tetrahedral Co clusters (**a**) **2**–**3** (Equation (1)) and (**b**) **4** (Equation (2)) from the reaction of Co_2_(CO)_8_ with [Cr(CO)_5_(PH_3_)], [W(CO)_5_(AsH_3_)] and [Fe(CO)_4_(PH_3_)], respectively, in toluene at room temperature.

[W(CO)_5_(AsH_3_)] (**1**) is synthesized in good yields from the reaction of [W(CO)_6_] with As(SiMe_3_)_3_ under UV irradiation with subsequent methanolysis of the reaction mixture. This compound was already reported by Fischer et al. [24], who used, however, AsH_3_ as an arsenic source. Herein, we completed the analytical data by ^13^C{^1^H} NMR as well as single crystal structure analysis (for further information see ESI). The reaction of [Cr(CO)_5_(PH_3_)], [W(CO)_5_(AsH_3_)] (**1**) or [Fe(CO)_4_(PH_3_)] with one equivalent of [Co_2_(CO)_8_] in toluene at room temperature allowed for the synthesis of cobalt clusters with the general formula [{M(CO)_n_}{Co(CO)_3_}_3_E] (n = 5, M = Cr, E = P (**2**), M = W, E = As (**3**); n = 4, M = Fe, E = P (**4**)) (Scheme 1, Equations (1) and (2)). Vahrenkamp et al. synthesized **2** using similar starting materials but in benzene instead of toluene [25]. They obtained compound **2** in 22% yield while the yield could be improved to 44% under our reaction conditions. Additionally, **2** was only characterized by IR spectroscopy and elemental analysis. Herein, we completed its analytical data (solution and solid-state IR; ^1^H, ^13^C{^1^H} and ^31^P{^1^H} NMR; EI-MS; EA) and characterized it by X-ray structural analysis. In the same study of Vahrenkamp, the P-analog of cluster **3** was synthesized from [W(CO)_5_(PH_3_)] and [Co_2_(CO)_8_]. Vizi-Orosz reported cluster **4** by the reaction of [Fe_2_(CO)_9_] with [Co_3_P(CO)_9_] but in very low yields (ca. 1%) [14] and characterized it only by EA. In our case, it was possible to prepare **4** in a different way (Equation (2)), resulting in improved yields (24%) and obtaining complete analytic data. In the ^31^P{^1^H} NMR spectrum of **2**, a singlet at 697.9 ppm (ω_1/2_ = 113 Hz) was detected while a broad signal at 677.5 ppm (ω_1/2_ = 175 Hz) was observed in that of **4**. The ^13^C{^1^H} NMR spectra of **2** and **3** show typical broad signals at 197.5 ppm (ω_1/2_ = 43 Hz) (**2**) and 197.0 ppm (ω_1/2_ = 35 Hz) (**3**) for the carbonyl carbon atoms, which belong to the Co_3_E tetrahedra (E = P (**2**), As (**3**)). Additionally, a doublet at 214.7 ppm with a C–P coupling constant (13.7 Hz) (**2**) and a singlet at 195.4 ppm (**3**) were observed. In the ^13^C{^1^H} NMR spectrum of **4**, a doublet at 212.0 ppm was detected with a C–P coupling constant of 18.8 Hz belonging to the cis carbonyl ligands. The signal for the trans carbonyl ligand could not be observed, while a broad signal at 197.0 ppm (ω_1/2_ = 46 Hz) for the carbonyl carbon atoms at the Co_3_P tetrahedron was detected. The broad signals in the multinuclear NMR spectra of compounds **2**–**4** originate from the coupling with the ^59^Co nucleus with a spin of 7/2 and 100% abundance. The EI mass spectra of **3**–**5** exhibit the molecular ion peak as well as peaks showing the successive loss of all carbonyl ligands. Furthermore, the loss of a chromium atom as well as a cobalt atom (**2**), two cobalt atoms (**3**) and one iron atom (**4**) were detected (for further information see Materials and Methods section and ESI).

**Figure 1:**
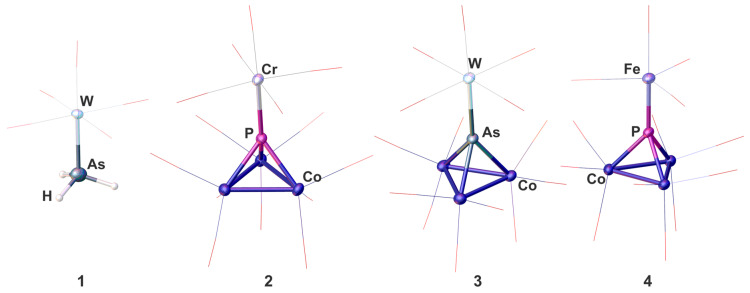
Molecular structure of compound **1** and the tetrahedral Co clusters **2**–**4** in the solid state (anisotropic displacement parameters (ADPs) are given at the 50% probability level). CO ligands are depicted in the wireframe model for clarity.

Compound **1** is obtained through sublimation as colorless crystals. It crystallizes in the monoclinic space group *P*2_1_/c. The central tungsten atom possesses an octahedral coordination sphere with five CO ligands and one As atom. Crystals of compounds **2**–**4** were obtained from concentrated n-hexane solutions stored at −25 °C. They crystallize in the monoclinic space groups *Cc* (**2**) and *P*2_1_/*n* (**4**) and the triclinic space group *P*ī (**3**). Their molecular structures reveal spiked tetrahedral molecules in which the central structural motifs “Co_3_P (**2**, **4**) or a Co_3_As (**3**)” are slightly distorted tetrahedranes that coordinate to the corresponding transition metal carbonyl fragment ([Cr(CO)_5_] (**2**), [W(CO)_5_] (**3**), [Fe(CO)_4_] (**4**)) via the lone pairs of the pnictogen atoms (Figure 1). The Co–Co distances in **2**–**4** (2.536(1)–2.569(1) Å) are in the range of Co–Co single bonds reported, e.g., for [CpCo(µ-PPh)]_2_ (2.56 Å) [26]. Accordingly, the tetrahedral cores of **2**–**4** each possess 48 cluster valence electrons (CVE) and can be described as closo compounds according to the Wade–Mingos rules.

### 2.2. Synthesis and X-ray Structures of the Cobalt Clusters [Co_8_(CO)_16_(µ-CO)_4_P] (***5***) and [{Co_4_(CO)_11_}{Co(CO)_3_}_3_P] (***6***)

**Scheme 2:**
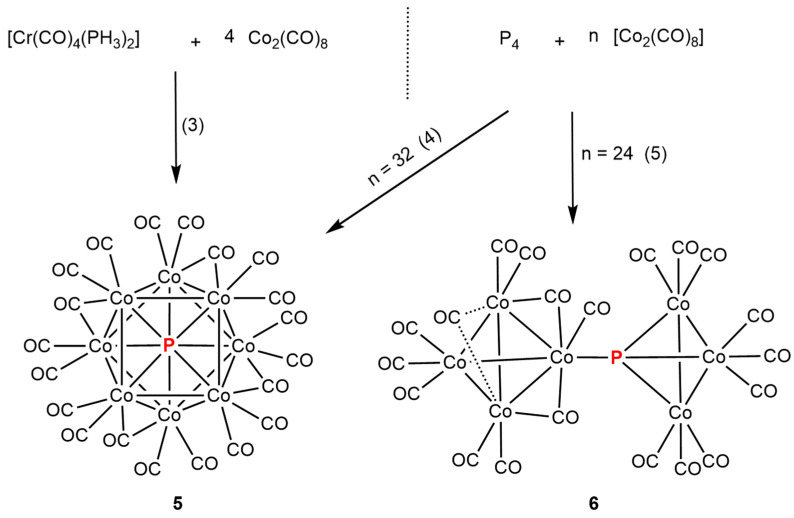
Synthesis of compound **5** from the reaction of Co_2_(CO)_8_ with Cr(CO)_4_(PH_3_)_2_] in toluene at room temperature (Equation (3)) and with P_4_ in hexane at −40 °C (Equation (4)). Synthesis of compound **6** from a similar reaction using 24 eq. of Co_2_(CO)_8_ in toluene at −100 °C (Equation (5)).

By using [Cr(CO)_4_(PH_3_)_2_] as a phosphorus source in the reaction with four equivalents of [Co_2_(CO)_8_] in toluene at room temperature, a new cobalt carbonyl cluster with an interstitial phosphorus atom [Co_8_(CO)_16_(µ-CO)_4_P] (**5**) is obtained in 6% yield (Scheme 2, Equation (3)). In addition to compound **5** and [Co_4_(CO)_12_], another cobalt phosphorus cluster [Co_8_(CO)_18_(µ-CO)(P)_2_] (**A**) is formed, previously obtained by our group from the reaction of [Co_2_(CO)_8_] with [W(CO)_4_(PH_3_)_2_] [27]. Another way to synthesize **5** in a slightly better yield (9%) is the reaction of P_4_ with an excess of [Co_2_(CO)_8_] (1:32 stoichiometry, n-hexane, Equation (4)). However, when the stoichiometry of P_4_ and [Co_2_(CO)_8_] is changed to 1:24 under slightly different reaction conditions, the cluster [{Co_4_(CO)_11_}{Co(CO)_3_}_3_P] (**6**) is formed instead of **5** (Scheme 2, Equation (5)).

**Figure 2:**
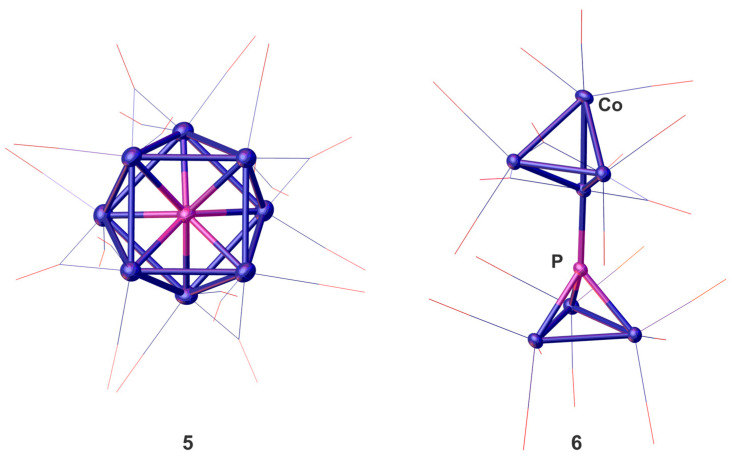
Molecular structure of the cluster compounds **5** and **6** in the solid state (atoms are given at 50% probability level). CO ligands are depicted in the wireframe model.

Single crystals of **5** were obtained from a dichloromethane solution or an n-hexane solution stored at −25 °C. It crystallizes in the monoclinic space group *P*2_1_/*n*. The central structural feature of **5** can be described as a quadratic antiprism formed by eight Co atoms and an interstitial P atom at the center with a total of 117 CVEs (Figure 2). Each Co atom in **5** is bound to four other Co atoms and to the central P atom with angles between the various Co-–Co and Co–P bonds being close to 60 and 90°. Additionally, each Co atom is coordinated by two terminal and one bridging CO ligand. Accordingly, four bridging and sixteen terminal CO ligands exist in **5**. The overall average Co–CO bond distances for bridging and terminal CO ligands are 1.961(8) and 1.810(8) Å, respectively. Similarly, two classes of Co–Co bonds can be distinguished: (a) shorter Co–Co bonds between Co atoms connected via bridging CO ligands range between 2.592(2) and 2.613(2) Å, and (b) longer Co–Co bonds between neighboring Co atoms with no bridging CO ligands ranging between 2.651(1) and 2.787(2) Å. The Co–P lengths are between 2.216(2) and 2.245(2) Å. DFT calculations at the r^2^SCAN-3c level reproduce the experimental geometry determined by X-ray diffractions well in both doublet and quartet sextet spin states. The spin states do not have a considerable influence on the geometry, which suggests that the spin density is located in a nonbonding Co orbital. Indeed, the spin density in the doublet spin state is evenly distributed on four cobalt atoms, with only small contributions from the other atoms (Figure S12). Energetically, the doublet spin state is 46 kJ·mol^−1^ more stable than the quartet spin state. This is not unexpected since the strong field ligands, for instance, CO, prefer the low spin configurations on the metal centers.

Compound **6** was obtained as black blocks from a concentrated toluene solution at −25 °C and crystallized in the triclinic space group *P*ī. Cluster **6** is composed of two slightly distorted tetrahedra, [P{Co(CO)_3_}_3_] and [Co_4_(CO)_11_], which are linked together via the coordination of the lone pair of the phosphorus atom in the former to a cobalt atom in the latter (Figure 2). Interestingly, the [P{Co(CO)_3_}_3_] tetrahedron contains only terminal CO ligands (three per cobalt atom) while the [Co_4_(CO)_11_] fragment contains eight terminal and three bridging CO ligands. The [P{Co(CO)_3_}_3_] tetrahedron in **6** shows a structural motif similar to that of compound [{W(CO)_5_}{Co(CO)_3_}_3_P] reported by Vahrenkamp et al. [25], and the structure of compound **6** as a whole is comparable to that of the clusters [{Co_3_(CO)_9_}(μ_4_-P){Co_3_(μ-CO)_3_(CO)_5_}(μ_3_-CR)] (R = CH_3_, *t*Bu, iPr) recently reported by our group from the reaction of the phopsphaalkynes RCP (R = CH_3_, *t*Bu, iPr) with Co_2_(CO)_8_ [28]. The Co–Co (2.548(6)–2.560(6) Å) bond distances in the [P{Co(CO)_3_}_3_] moiety are longer than those reported for [{W(CO)_5_}{Co(CO)_3_}_3_P] and comparable to those found in clusters **2**–**4**. The Co–Co (2.444(6)–2.529(6) Å) bond lengths in the [Co_4_(CO)_11_] fragment are, as expected, shorter due to the presence of bridging CO ligands. The Co–P bond lengths (2.171(9)–2.183(9) Å) in the [P{Co(CO)_3_}_3_] fragment are slightly shorter than those found in **2** (2.177(3)–2.191(3) Å) and longer than those found in **4** (2.162(2)–2.169(2) Å). The Co atoms in **6** are bound through metal–metal bonds and fulfill the 18-valence electron rule. Thus, the {Co(CO)_3_}_3_P part in **6** can be regarded as a closo cluster with 48 CVE with the lone pair at P engaging in dative bonding to the [Co_4_(CO)_11_] tetrahedron, which is best described as a nido cluster (tetrahedron derived from trigonal bipyramid) with 60 CVE (4 × 9 (Co) + 11 × 2 (CO) + 2 (P{Co(CO)_3_}_3_)).

### 2.3. Synthesis and X-ray Structures of the Cobalt Clusters [Co_9_(CO)_24_(µ_4_-P)_3_] (***7***) and [Co_9_(CO)_21_(µ_5_-P)_3_] (***8***)

**Scheme 3:**
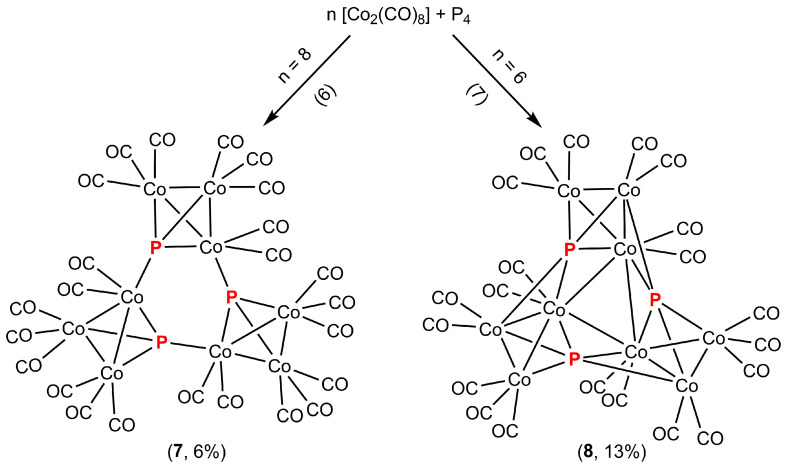
Synthesis of the cyclic clusters **7** and **8** from the reaction of P_4_ with eight eq. of Co_2_(CO)_8_ in toluene at room temperature (Equation (6)) and six eq. of Co_2_(CO)_8_ in hexane at room temperature (Equation (7)), respectively.

Subsequently, we focused on the reaction of [Co_2_(CO)_8_] with P_4_ using various ratios and reaction conditions. Reactions using eight equivalents of [Co_2_(CO)_8_] in toluene (Scheme 3, Equation (6)) and six equivalents of [Co_2_(CO)_8_] in hexane (Scheme 3, Equation (7)) at room temperature resulted in the compounds [Co_9_(CO)_24_(µ_4_-P)_3_] (**7**) and [Co_9_(CO)_21_(µ_5_-P)_3_] (**8**), respectively. Both compounds consist of three [Co_3_P(CO)_9_] fragments that have lost three (**7**) or six (**8**) CO ligands with subsequent formation of new Co–Co and Co–P bonds. Markó et al. [29] isolated a compound with a molecular formula similar to **7** and proposed a cyclic structure as in **7** based on a similar reaction protocol used to obtain the analogous cyclic As-Co trimer. Herein, the formation of compound **7** is evidenced by X-ray crystallography. As for compound **8**, however, the rather poor crystal quality only allowed for the collection of an incomplete data set proving the structure of **8**, but not for structural analysis in detail.

**Figure 3:**
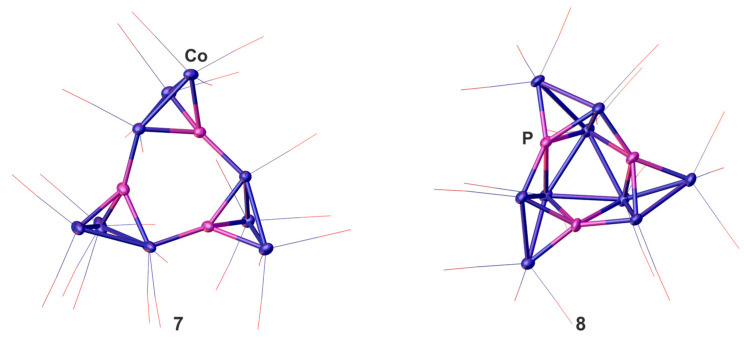
Molecular structures of the cluster compounds **7** and **8** in the solid state (ADPs are given at the 50% probability level). CO ligands are depicted in the wireframe model for clarity.

Compounds **7** and **8** are isolated as dark violet (**7**) and black block-shaped (**8**) crystals from n-hexane solutions stored at −25 °C and 8 °C, respectively. Cluster **7** crystallizes in the monoclinic space group *P*2_1_/*n* while **8** crystallizes in the orthorhombic space group *P*2_1_2_1_2_1_. Compound **7** consists of three [PCo_3_(CO)_8_] tetrahedranes that are connected together via dative Co–P bonds, thus forming a cyclic trimer with a six-membered P_3_Co_3_ central ring (Figure 3). Compound **8** consists of three [PCo_3_(CO)_7_] units connected to one another by two Co–P bonds and one Co–Co bond. Theoretically, cluster **8** could have been formed from **7** by CO elimination, which could, however, not have been proven experimentally due to the insufficient solubility of **7** in common organic solvents. The Co–Co (2.530(8)–2.584(8) Å) bond lengths in **7** are generally comparable to those of **2**–**4** and the Co–P (2.153(1)–2.200(1) Å) bond lengths are comparable to those of **2**. All CO ligands in **7** and **8** are terminal ones with Co–CO lengths ranging between 1.775(7) and 1.898(1) Å, e.g., for **7**. Overall, compound **7** amounts to 144 CVEs from three [PCo_3_(CO)_8_] closo tetrahedra each possessing 48 CVEs. In compound **8**, the overall CVEs amount to 138 (9 × 9 (Co) + 21 × 2 (CO) + 3 × 5 (P)).

### 2.4. Synthesis and X-ray Structures of the Cobalt Clusters [Co_10_(CO)_24_(µ_3_-P)_2_(µ_6_-P_2_)(µ-CO)_2_] (***9***) and [Co_15_(µ_6_-P)_6_(µ_12_-Co)(CO)_30_] (***10***)

**Scheme 4:**
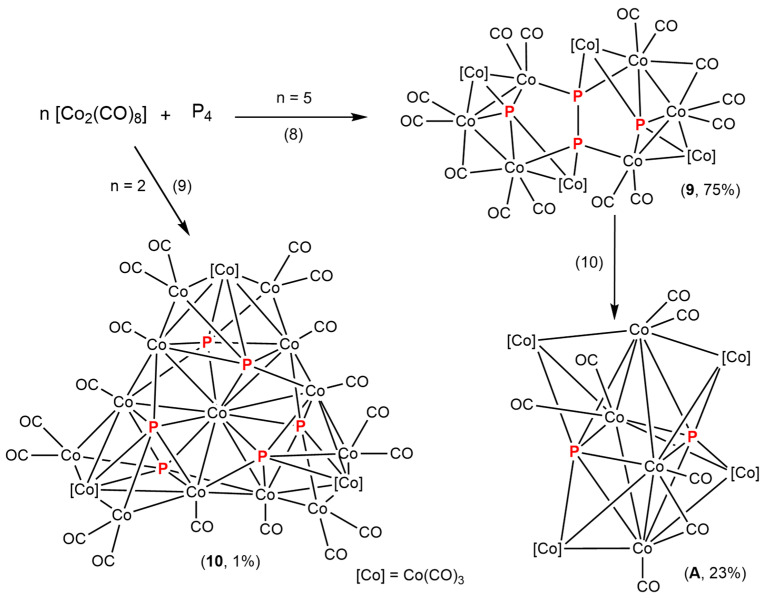
Synthesis of compounds **9** and **10** from the reaction of P_4_ with five eq. of Co_2_(CO)_8_ from toluene/n-hexane layering at room temperature (Equation (8)) and two eq. of Co_2_(CO)_8_ in toluene at room temperature (Equation (9)), respectively. Thermal transformation of **9** to cluster **A** in boiling toluene (Equation (10)).

When a solution of five equivalents of [Co_2_(CO)_8_] in toluene is layered with a solution of one equivalent P_4_ in n-hexane, black crystals of [Co_10_(CO)_24_(µ_3_-P)_2_(µ_5_-P)_2_(µ-CO)_2_] (**9**) are obtained in excellent yields of 75% (Scheme 4, Equation (8)). When the components are instead employed in stirring reactions, using two equivalents of [Co_2_(CO)_8_] with one equivalent of P_4_ in toluene at room temperature, cluster **9** is also obtained but in very low yields (1%). Besides **9**, another cluster [Co_15_(µ_6_-P)_6_(µ_12_-Co)(CO)_30_] (**10**) can be isolated from the same reaction mixture after a long crystallization time in very low yields of 1% (Scheme 4, Equation (9)). Interestingly, compound **9** can be further refluxed in toluene to give the cluster [Co_8_(CO)_18_(μ-CO)(P)_2_] (**A**) [27] in good yields (22%) as a thermally stable cluster via a new synthetic pathway (Scheme 4, Equation (10)).

**Figure 4:**
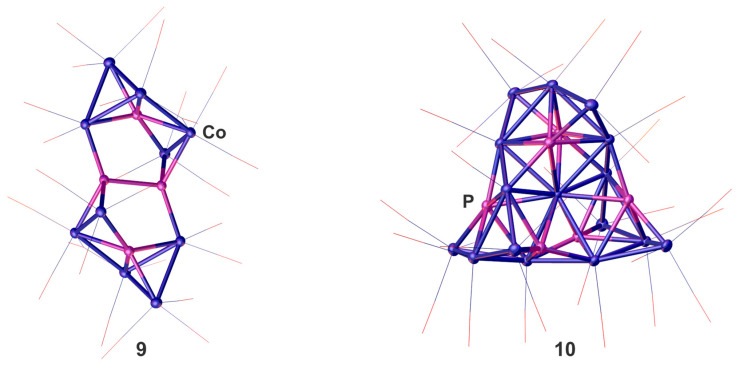
Molecular structure of the cluster compounds **9** and **10** in the solid state (ADPs are given at the 50% probability level). CO ligands are depicted in the wireframe model for clarity.

Compound **9** is isolated as black plates and crystalizes in the monoclinic space group *P*2_1_/*c*. Also, black rods of **9**·C_6_H_14_ can be obtained from a concentrated n-hexane solution crystallizing in the tetragonal space group *I*4_1_/*a*. The cluster core in **9** is constructed of ten Co and four P atoms with 24 terminal and two bridging CO ligands being coordinated to the Co atoms (Figure 4). It can also be described as two Co_5_P fragments that are connected together by a P_2_ moiety located at its inversion center with a P–P bond length of 2.265(1) Å, which is slightly longer but still at the upper limit of a single P–P bond (2.212(2) Å) [30]. Each Co atom in **9** fulfills the 18-valence electron rule with a total of 162 CVEs (10 × 9 (Co) + 26 × 2 (CO) + 4 × 5 (P)).

**Figure 5:**
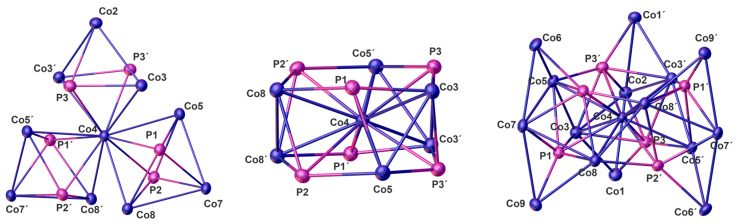
Molecular structure details of the core of cluster **10** without CO ligands. (**Left**): central distorted Co_4_P_2_ octahedra; (**middle**): central hexagonal antiprism-like structural motif; (**right**): whole core structure.

Crystals of **10**•2C_7_H_8_ crystallize in the monoclinic space group *C*2/*c*. This cluster is composed of 16 Co and 6 P atoms (Figure 4). Besides the central cobalt atom, which is located in the middle of the cluster (Co4), every Co atom is coordinated by one, two or three terminal CO ligands from a total of thirty CO ligands present in **10**. Each P atom in **10** is surrounded by six cobalt atoms, revealing the coordination number six. A simplification of the structural details of **10** is depicted in Figure 5. The core of this compound is composed of three distorted Co_4_P_2_ octahedra with Co4 being the center of their intersection (one of them for example is composed of these atoms: Co4, Co5′, Co8′, P2′, P1′; Co7′, Figure 5 left). This central octahedral structural motif is similar to the central structural motif in the reported clusters **A** and [Co_10_(CO)_18_(μ-CO)_6_P_2_] (**B**) [27]. When considering the Co–Co bonds between the atoms Co3 and Co5, Co3′ and Co5′ and Co8 and Co8′, another central structural motif becomes apparent. This motif forms a hexagonal antiprism, consisting of six cobalt and six phosphorus atoms with an interstitial cobalt atom (Figure 5 middle). Finally, the addition of the rest of the Co atoms completes the molecular structure of **10** (Figure 5 right). Within **10**, various Co_3_P tetrahedra are found (e.g., Co7, Co8, Co9, P1) with Co–Co bond lengths ranging between 2.5029(8) and 2.6735(8) Å and, therefore, lie in the range of those discussed for clusters **2**–**9**. For each Co atom in **10**, the 18-valence electron rule is fulfilled with a total of 234 CVEs for the core (16 × 9 (Co) + 30 × 2 (CO) + 6 × 5 (P)).

### 2.5. New Synthetic Protocol for the Synthesis of the Cobalt Clusters [Co_9_(CO)_24_(µ_4_-As)_3_] (***11***) and [{Co_4_(CO)_11_}{Co(CO)_3_}_3_As] (***12***)

**Scheme 5:**
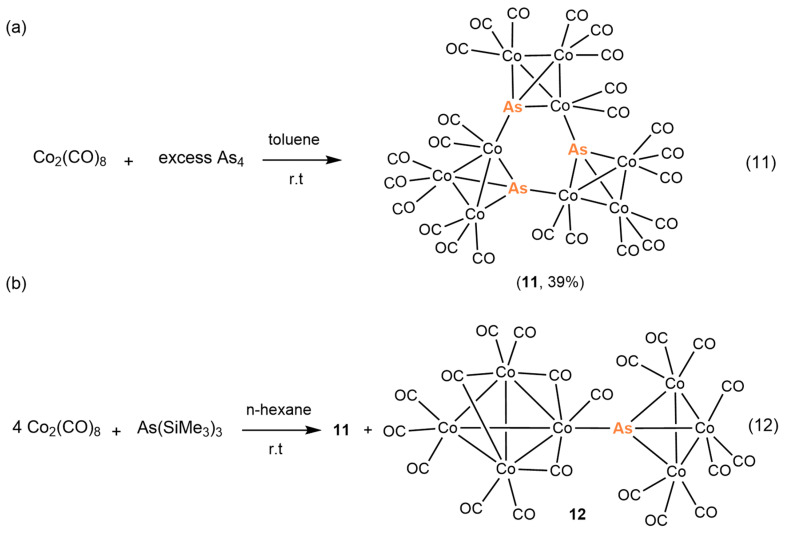
Synthesis of the cyclic clusters (**a**) **11** from the reaction of Co_2_(CO)_8_ with As_4_ in toluene at room temperature (Equation (11)), and (**b**) **12** from the reaction of Co_2_(CO)_8_ with As(SiMe_3_)_3_ in n-hexane at room temperature (Equation (12)).

Finally, [Co_2_(CO)_8_] was reacted with the arsenic sources yellow arsenic As_4_ (Scheme 5, Equation (11)) and As(SiMe_3_)_3_ (Scheme 5, Equation (12)), respectively. The first reaction afforded the cluster [Co_9_(CO)_24_(µ_4_-As)_3_] (**11**) good yields (39%). Markó et al. [29] and the groups of Mackay and Nicholson [12] reported the synthesis of **11** in 8% and 17% yields, respectively. The former described it as a “cyclic trimer” of the trigonal pyramidal cluster [AsCo_3_(CO)_9_]. This compound was obtained together with AsCo_3_(CO)_9_ and As_2_Co_2_(CO)_6_ from the reaction of Na[Co(CO)_4_] with AsCl_3_. Using our new strategy, **11** was obtained in better yields, and its full analytical characterization was performed. From the reaction with As(SiMe_3_)_3_, besides product **11**, it was possible to isolate the cobalt arsenic cluster [{Co_4_(CO)_11_}{Co(CO)_3_}_3_As] (**12**) in low yields (1%), which had already been reported by Huttner et al. from the reaction of [Co_2_(CO)_8_] with [Cr(CO)_5_(AsPhH_2_)] [31]. Interestingly, we were able to obtain compounds **11** and **12** as single crystals but in polymorphs differing from those of the initially reported ones.

Compounds **6**, **7**, **8**, **11** and **12** are soluble in toluene and THF while compounds **5**, **9** and **10** are nearly insoluble in common organic solvents. In the EI mass spectra of **6**, **7**, **8**, **11** and **12**, the molecular ion peak as well as the peaks showing the successive loss of all carbonyl ligands are detected (for further information see Section 3 and ESI). Thus, the fragments at *m*/*z* = 443.6 for **6** and 487.6 for **12** can be observed, which shows the remaining cluster cores “Co_7_P” or “Co_7_As”. For compounds **7**, **8** and **11**, peaks contributing to the cluster cores “Co_9_P_3_” at *m*/*z* = 623.4 or “Co_9_As_3_” at *m*/*z* = 755.2 are detected. The ^31^P{^1^H} NMR spectra of **6**, **7**, and **8** show broad signals at 666.4 (ω_1/2_ = 114 Hz), 667.3 (ω_1/2_ = 362 Hz) or 658.2 ppm (ω_1/2_ = 205 Hz). In the ^31^P{^1^H} MAS NMR spectrum of **9**, a broad signal at 684 ppm (ω_1/2_ = 2800 Hz) can be detected. Due to the insolubility of some compounds and the high sensitivity of most of the clusters, it was not possible to characterize all of them completely. As the cluster cores of compounds **2**–**12** are mainly surrounded by CO ligands that can only be readily released according to the mass spectra, clusters **2**–**12** could be used as potential single source precursors for the synthesis of Co_x_P_y_ and/or Co_x_As_y_ nanoparticles with variable ratios of the elements.

## 3. Materials and Methods

### 3.1. General Information

All manipulations were carried out under a dry argon or dinitrogen atmosphere using glovebox or standard Schlenk techniques. The solvents were dried using standard procedures and were freshly distilled prior to use. The starting materials [Cr(CO)_5_(PH_3_)] [32], [Fe(CO)_4_(PH_3_)] [33], [Cr(CO)_4_(PH_3_)_2_] [34], As_4_ [35] and As(SiMe_3_)_3_ [36] were synthesized according to literature procedures. [Cr(CO)_6_], [W(CO)_6_] and [Co_2_(CO)_8_] were purchased from Merck (Darmstadt, Germany) and used without further purification. The NMR spectra were recorded on a Bruker Advance 400 (Billerica, MA, USA, ^1^H, 400.132 MHz; ^31^P, 161.975 MHz, ^13^C, 100.613 MHz) referenced to external SiMe_4_ (^1^H) or H_3_PO_4_ (^31^P), respectively. ^31^P{^1^H} MAS NMR spectra were recorded with a Bruker Advance 300 spectrometer equipped with a double resonance 2.5 mm MAS probe. The spectra were acquired at MAS rotation frequencies of 30 and 34 kHz, a 90° pulse length of 2.3 μs, and with relaxation delays between 120 and 450 s. Mass spectra were recorded on a Finnnigan MAT SSQ 710 A (EI) spectrometer (Scientific Instrument Services, Palmer, MA, USA). IR spectra were measured with a Varian FTS-800 spectrometer (Palo Alto, CA, USA). Elemental analyses (CHN) were determined on a Vario EL III instrument (Elementar Analysensysteme GmbH, Langenselbold, Germany).

### 3.2. Synthesis and Characterization of Clusters ***2**–**12***

#### 3.2.1. Synthesis and Characterization of Clusters **2** and **3**

To a stirred solution of [Cr(CO)_5_(PH_3_)] (69.0 mg, 0.3 mmol) or [W(CO)_5_(AsH_3_)] (**1**) (121 mg, 0.3 mmol) in toluene, a solution of [Co_2_(CO)_8_] (103 mg, 0.3 mmol) in toluene was added. The crude mixture was then stirred for 24 h at room temperature. The solvent was removed under reduced pressure, and the dark residue was extracted with 10 mL n-hexane and stored at −25 °C. After one day, dark red crystals of **2** and violet black crystals of **3**, respectively, were obtained. Yield of **2**: (1.11 g, 57%). Yield of **3**: 86.2 mg (44%) (**2**); 59.6 mg (24%) (**3**). IR (KBr): ῦ/cm^−1^ = ν_CO_: 2113 (s), 2085 (vs sh), 2053 (vs), 2031 (s), 2000 (w), 1990 (m), 1963 (s sh), 1944 (vs) (**2**); IR (toluene): ῦ/cm^−1^ = ν_CO_: 2111 (w), 2062 (vs), 2043 (vw), 2034 (w), 1977 (sh), 1956 (m) (**2**); IR (KBr): ῦ/cm^−1^ = ν_CO_: 2110 (w), 2079 (m), 2057 (vs), 2038 (m), 2032 (m), 2018 (w), 1992 (w), 1937 (vs) (**3**). ^31^P{^1^H} NMR (161.975 MHz, C_6_D_6_): *δ*[ppm] = 697.9 (br s, *ω*_1/2_ = 113 Hz). ^13^C{^1^H} NMR (100.613 MHz, C_6_D_6_): *δ* = 197.5 (br s, *ω*_1/2_ = 43 Hz, (Co(CO)_3_)_3_), 214.7 (d, ^2^*J*_CP_ = 13.7 Hz, Cr(CO)_4_) (**2**); 197.0 (br s, *ω*_1/2_ = 35 Hz, (Co(CO)_3_)_3_), 195.4 (s, W(CO)_4_) (**3**). EI-MS (70 eV): *m*/*z* (%) = 651.7 (25) [M^+^], 623.6 (21) [M^+^*–CO*], 567.6 (6) [M^+^*–3 CO*], 539.7 (17) [M^+^*–4 CO*], 511.6 (42) [M^+^*–5 CO*], 483.7 (95) [M^+^*–6 CO*], 455.7 (68) [M^+^*–7 CO*], 427.7 (52) [M^+^*–8 CO*], 399.7 (58) [M^+^*–9 CO*], 371.7 (59) [M^+^*–10 CO*], 343.7 (59) [M^+^*–11 CO*], 315.7 (59) [M^+^*–12 CO*], 287.7 (53) [M^+^*–13 CO*], 259.7 (100) [M^+^*–14 CO*], 207.7 (53) [M^+^*–14 CO–Cr*], 148.8 (38) [M^+^*–14 CO–Cr–Co*] (**2**); 827.8 (24) [M^+^], 799.9 (34) [M^+^*–CO*], 743.8 (4) [M^+^*–3 CO*], 715.8 (13) [M^+^*–4 CO*], 687.8 (69) [M^+^*–5 CO*], 659.9 (100) [M^+^*–6 CO*], 603.8 (57) [M^+^*–8 CO*], 575.9 (80) [M^+^*–9 CO*], 547.8 (49) [M^+^*–10 CO*], 519.8 (55) [M^+^*–11 CO*], 491.8 (63) [M^+^*–12 CO*], 463.8 (63) [M^+^*–13 CO*], 435.9 (99) [M^+^*–14 CO*], 376.8 (76) [M^+^*–14 CO–Co*], 317.9 (28) [M^+^*–14 CO–2 Co*] (**3**). Elemental analysis, calcd. for Co_3_PCr(CO)_14_ (651.64 g/mol): C, 25.79. Found C, 25.72 (**2**). Elemental analysis, calcd. for Co_3_AsW(CO)_14_ (827.60 g/mol): C, 20.32. Found C, 20.22 (**3**).

#### 3.2.2. Synthesis and Characterization of Cluster **4**

A solution of [Fe(CO)_4_(PH_3_)] (60.6 mg, 0.3 mmol) in toluene was added to a solution of [Co_2_(CO)_8_] (103 mg, 0.3 mmol) in toluene and stirred for 18 h. After removing the solvent under reduced pressure, the black residue was dissolved in 10 mL hexane and filtrated. Brown blocks of **4** were obtained within a few hours by storing the hexane solution at −25 °C. Yield: 45.2 mg (24%). IR (KBr): ῦ/cm^−1^ = ν_CO_: 2115 (m), 2080 (s sh), 2055 (vs), 2033 (s), 2020 (m), 1984 (m), 1952 (m), 1941 (s); IR (hexane): ῦ/cm^−1^ = ν_CO_: 2112 (w), 2089 (w br), 2069 (vs), 2058 (s), 2039 (w), 1986 (m br), 1960 (w), 1932 (w br). ^31^P{^1^H} NMR (161.975 MHz, C_6_D_6_): *δ* [ppm] = 677.5 (br s, *ω*_1/2_ = 175 Hz). ^13^C{^1^H} NMR (100.613 MHz, C_6_D_6_): *δ* = 197.0 (br s, *ω*_1/2_ = 46 Hz, (Co(CO)_3_)_3_), 212.0 (d, ^2^*J*_CP_ = 18.8 Hz, Fe(CO)_3_). EI-MS (70 eV): *m*/*z* (%) = 627.7 (22) [M^+^], 599.8 (20) [M^+^*–CO*], 571.8 (39) [M^+^*–2 CO*], 543.8 (19) [M^+^*–3 CO*], 515.7 (9) [M^+^*–4 CO*], 487.8 (21) [M^+^*–5 CO*], 459.8 (100) [M^+^*–6 CO*], 431.8 (75) [M^+^*–7 CO*], 403.8 (50) [M^+^*–8 CO*], 375.8 (55) [M^+^*–9 CO*], 347.8 (50) [M^+^*–10 CO*], 319.8 (44) [M^+^*–11 CO*], 291.8 (42) [M^+^*–12 CO*], 263.9 (92) [M^+^*–13 CO*], 207.9 (36) [M^+^*–13 CO–Fe*]. Elemental analysis, calcd. for Co_3_PFe(CO)_13_ (627.64 g/mol): C, 24.87. Found C, 24.96.

#### 3.2.3. Synthesis and Characterization of Cluster **5**

Method 1: A solution of [Cr(CO)_4_(PH_3_)_2_] (232 mg, 1 mmol) in 50 mL toluene was added to a solution of [Co_2_(CO)_8_] (1368 mg, 4 mmol) in 50 mL toluene and stirred for seven days at room temperature. The solvent was then completely removed under reduced pressure, and the crude product was dissolved in hexane and filtrated. This filtrate contained [Co_4_(CO)_12_]. The residue in the frit was extracted with dichloromethane and found to contain the product [Co_8_(CO)_18_(µ-CO)_4_P] (**5**). The remaining dark red residue was collected and dissolved in dichloromethane overnight. After filtration and reducing the solution under reduced pressure up to 30 mL, crystals of **5** were obtained upon storing at −25 °C after two weeks. Yield: 63.7 mg (6%).

Method 2: A stirred solution of P_4_ (3 mg, 0.025 mmol) and [Co_2_(CO)_8_] (274 mg, 0.8 mmol) in 30 mL cold hexane at −40 °C was warmed up to room temperature under stirring. After further stirring for two days, the reaction mixture was filtrated and stored at room temperature. Crystals of **5** were obtained within two weeks. Yield: 19.0 mg (9%).

#### 3.2.4. Synthesis and Characterization of Cluster **6**

P_4_ (4 mg, 0.03 mmol) and [Co_2_(CO)_8_] (274 mg, 0.8 mmol) were dissolved in 40 mL toluene, cooled to −100 °C and stirred for 30 min. The reaction mixture was then warmed up to room temperature and further stirred for six days. The reaction mixture was filtrated and stored at −25 °C for three months from which black blocks of **6** were obtained. Yield: ~1%. IR (KBr): ῦ/cm^−1^ = ν_CO_: 2053 (vs), 2037 (vs), 1896 (m), 1848 (s). ^31^P{^1^H} NMR (161.975 MHz, C_6_D_6_): *δ* [ppm] = 666.4 (br s, *ω*_1/2_ = 114 Hz). EI-MS (70 eV): *m*/*z* (%) = 1003.4 (3) [M^+^], 947.5 (27) [M^+^*–2 CO*], 919.4 (35) [M^+^*–3 CO*], 891.4 (21) [M^+^*–4 CO*], 863.3 (17) [M^+^*–5 CO*], 835.3 (65) [M^+^*–6 CO*], 807.4 (79) [M^+^*–7 CO*], 779.4 (54) [M^+^*–8 CO*], 751.4 (57) [M^+^*–9 CO*], 723.9 (49) [M^+^*–10 CO*], 695.5 (43) [M^+^*–11 CO*], 667.4 (59) [M^+^*–12 CO*], 639.5 (59) [M^+^*–13 CO*], 611.5 (57) [M^+^*–14 CO*], 583.6 (55) [M^+^*–15 CO*], 555.4 (65) [M^+^*–16 CO*], 527.5 (58) [M^+^*–17 CO*], 499.6 (1) [M^+^*–18 CO*], 471.6 (1) [M^+^*–19 CO*], 443.6 (3) [M^+^*–20 CO*].

#### 3.2.5. Synthesis and Characterization of Cluster **7**

A solution of P_4_ (47 mg, 0.38 mmol) and [Co_2_(CO)_8_] (1026 mg, 3.0 mmol) in 40 mL toluene was stirred for ten days at room temperature. After removing the solvent under reduced pressure, the residue was dissolved in hexane and filtrated. Black blocks of **7** were obtained after storage at 8 °C. Yield: 36.0 mg (6%). IR (KBr): ῦ/cm^−1^ = ν_CO_: 2034 (s br). ^31^P{^1^H} NMR (161.975 MHz, C_6_D_6_): *δ* = 658.2 (br s, *ω*_1/2_ = 205 Hz). EI-MS (70 eV): *m*/*z* (%) = 1211.2 (6) [M^+^], 1183.2 (23) [M^+^*–CO*], 1127.3 (11) [M^+^*–3 CO*], 1099.2 (3) [M^+^*–4 CO*], 1071.3 (16) [M^+^*–5 CO*], 1043.3 (36) [M^+^*–6 CO*], 1015.3 (42) [M^+^*–7 CO*], 987.3 (29) [M^+^*–8 CO*], 959.3 (24) [M^+^*–9 CO*], 931.4 (23) [M^+^*–10 CO*], 903.3 (24) [M^+^*–11 CO*], 875.3 (25) [M^+^*–12 CO*], 847.4 (25) [M^+^*–13 CO*], 819.4 (26) [M^+^*–14 CO*], 791.4 (23) [M^+^*–15 CO*], 763.3 (32) [M^+^*–16 CO*], 735.4 (27) [M^+^*–17 CO*], 707.4 (23) [M^+^*–18 CO*], 679.4 (28) [M^+^*–19 CO*], 651.4 (28) [M^+^*–20 CO*], 623.4 (100) [M^+^*–21 CO*]. Elemental analysis, calcd. for Co_9_P_3_C_21_O_21_ (1211.21 g/mol): C, 20.82. Found C, 21.06.

#### 3.2.6. Synthesis and Characterization of Cluster **8**

A solution of P_4_ (12 mg, 0.1 mmol) and [Co_2_(CO)_8_] (205 mg, 0.6 mmol) in 40 mL cold hexane at −40 °C was warmed up to room temperature under stirring. After further stirring for two days the reaction mixture was filtrated and stored at −25 °C from which dark violet crystals of **8** were obtained. Yield: 67.0 mg (13%). IR (KBr): ῦ/cm^−1^ = ν_CO_: 2050 (s br), 2038 (s sh). ^31^P{^1^H} NMR (161.975 MHz, C_6_D_6_): *δ* = 667.3 (br s, *ω*_1/2_ = 362 Hz). EI-MS (70 eV): *m/z* (%) = 1267.7 (8) [M^+^*–CO*], 1239.6 (3) [M^+^*–2 CO*], 1211.8 (19) [M^+^*–3 CO*], 1183.8 (15) [M^+^*–4 CO*], 1155.8 (2) [M^+^*–5 CO*], 1127.8 (16) [M^+^*–6 CO*], 1071.1 (18) [M^+^ *–8 CO*], 1043.2 (63) [M^+^*–9 CO*], 1015.2 (77) [M^+^*–10 CO*], 987.2 (44) [M^+^*–11 CO*], 959.2 (40) [M^+^*–12 CO*], 931.2 (41) [M^+^*–13 CO*], 903.2 (39) [M^+^*–14 CO*], 875.2 (42) [M^+^*–15 CO*], 847.2 (38) [M^+^*–16 CO*], 819.2 (40) [M^+^*–17 CO*], 791.2 (40) [M^+^*–18 CO*], 763.3 (41) [M^+^*–19 CO*], 679.3 (2) [M^+^*–22 CO*], 651.3 (3) [M^+^*–23 CO*], 623.3 (4) [M^+^*–24 CO*].

#### 3.2.7. Synthesis and Characterization of Cluster **9**

A solution of [Co_2_(CO)_8_] (274 mg, 0.8 mmol) in 25 mL toluene was slowly layered with a solution of P_4_ (20 mg, 0.16 mmol) in 25 mL hexane. Within five days, a few black crystals of **10** were obtained. The complete crystallization needed a further five weeks. Yield: 175 mg (75%). IR (KBr): ῦ/cm^−1^ = ν_CO_: 2110 (vw), 2064 (s sh), 2054 (s sh), 2043 (vs br), 2032 (s sh), 2025 (s sh), 2001 (m sh), 1958 (w sh). ^31^P{^1^H} NMR (161.975 MHz, C_6_D_6_): *δ* = 684.0 (br s, *ω*_1/2_ = 2768 Hz). Elemental analysis, calcd. for Co_10_P_4_C_26_O_26_ (1441.44 g/mol): C, 21.66. Found C, 21.64.

#### 3.2.8. Synthesis and Characterization of Cluster **10**

A solution of P_4_ (25 mg, 0.2 mmol) in 20 mL toluene was added to a solution of [Co_2_(CO)_8_] (137 mg, 0.4 mmol) in 20 mL toluene and stirred for two days at room temperature. On days three and four, a little vacuum was applied over the reaction mixture to remove the evolved CO from the reaction atmosphere, and the solution mixture was stirred for two more days. The reaction mixture was then filtrated and stored at −25 °C. After six months, a few crystals of **9** and **10** were obtained. Yield: ~1%. IR (KBr): ῦ/cm^−1^ = ν_CO_: 2080 (m sh), 2060 (vs), 2039 (s sh), 1994 (m).

#### 3.2.9. Synthesis and Characterization of Cluster **11**

Method 1: A freshly prepared solution of As_4_ (17 mg, 55 µmol, 3.67 mmol L^−1^) in toluene was added to a solution of [Co_2_(CO)_8_] (274 mg, 0.8 mmol) in 30 mL toluene under light exclusion and stirred for four days at room temperature. The reaction mixture was filtrated and concentrated to 25 mL under reduced pressure. The mixture was then stored at −25 °C from which black blocks of **11** were obtained within three weeks. Yield 24 mg (39%).

Method 2: As(SiMe_3_)_3_ (0.03 mL, 0.1 mmol) was added to a solution of [Co_2_(CO)_8_] (137 mg, 0.4 mmol) in hexane and stirred for two days at room temperature. The dark red solution was filtrated and stored at −25 °C. After one month, black rods of **11** were obtained. Yield: ~1%. IR (KBr): ῦ/cm^−1^ = ν_CO_: 2089 (s), 2070 (vs), 2026 (vs), 2055 (s sh), 2008 (s). EI-MS (70 eV): *m/z* (%) = 1427.1 (7) [M^+^], 1399.2 (35) [M^+^*–CO*], 1343.2 (11) [M^+^*–3 CO*], 1315.3 (17) [M^+^*–4 CO*], 1287.4 (3) [M^+^*–5 CO*], 1259.4 (12) [M^+^*–6 CO*], 1231.4 (47) [M^+^*–7 CO*], 1203.4 (100) [M^+^*–8 CO*], 1175.4 (80) [M^+^*–9 CO*], 1147.5 (57) [M^+^*–10 CO*], 1119.4 (74) [M^+^*–11 CO*], 1063.4 (76) [M^+^*–13 CO*], 1035.0 (23) [M^+^*–14 CO*], 1007.0 (23) [M^+^*–15 CO*], 979.0 (24) [M^+^*–16 CO*], 951.1 (28) [M^+^*–17 CO*], 923.0 (21) [M^+^*–18 CO*], 895.1 (21) [M^+^*–19 CO*], 867.1 (21) [M^+^*–20 CO*], 839.2 (21) [M^+^*–21 CO*], 811.1 (22) [M^+^*–22 CO*], 783.2 (21) [M^+^*–23 CO*], 755.2 (80) [M^+^*–24 CO*]. Elemental analysis, calcd. for Co_9_As_3_C_24_O_214_ (1427.04 g/mol): C, 20.19. Found C, 20.30.

#### 3.2.10. Synthesis and Characterization of Cluster **12**

A solution of As(SiMe_3_)_3_ (0.03 mL, 0.1 mmol) and [Co_2_(CO)_8_] (137 mg, 0.4 mmol) in 20 mL hexane was stirred for two days at room temperature. The solution was filtered and stored at −25 °C from which black rods of **12** were isolated besides crystals of **11,** which could be manually separated from each other in a glove box. Yield: ~1%. EI-MS (70 eV): *m/z* (%) = 1047.4 (14) [M^+^], 991.3 (61) [M^+^*–2 CO*], 963.4 (20) [M^+^*–3 CO*], 935.3 (19) [M^+^*–4 CO*], 907.4 (20) [M^+^*–5 CO*], 879.4 (60) [M^+^*–6 CO*], 851.4 (72) [M^+^*–7 CO*], 823.4 (86) [M^+^*–8 CO*], 795.5 (56) [M^+^*–9 CO*], 767.5 (60) [M^+^*–10 CO*], 739.6 (70) [M^+^*–11 CO*], 711.5 (72) [M^+^*–12 CO*], 683.6 (64) [M^+^*–13 CO*], 655.5 (4) [M^+^*–14 CO*], 627.6 (4) [M^+^*–15 CO*], 599.6 (5) [M^+^*–16 CO*], 543.8 (16) [M^+^*–18 CO*], 515.8 (8) [M^+^*–19 CO*], 487.6 (14) [M^+^*–20 CO*].

#### 3.2.11. Synthesis and Characterization of Cluster **A**

The cluster **9** (100 mg, 0.07 mmol) was dissolved in 20 mL toluene and refluxed for three hours. The suspension was filtrated and stored at −25 °C. After one month, black crystals of **A** were isolated. Yield: 20 mg (21.7%). IR (KBr): ῦ/cm^−1^ = ν_CO_: 2108 (m), 2088 (vs), 2052 (vs), 2033 (vs), 2023 (vs), 2000 (vs), 1991 (s), 1979 (vs), 1966 (vs), 1826 (m), 1800 (s). ^31^P{^1^H} NMR (161.975 MHz, C_6_D_6_): *δ* [ppm] = 474.6 (br s, *ω*_1/2_ = 738 Hz). EI-MS (70 eV): *m/z* (%) = 1065.3 (26) [M^+^], 1037.2 (27) [M^+^*–CO*], 1009.2 (9) [M^+^*–2 CO*], 981.2 (5) [M^+^*–3 CO*], 953.2 (25) [M^+^*–4 CO*], 925.2 (63) [M^+^*–5 CO*], 897.4 (57) [M^+^*–6 CO*], 869.4 (50) [M^+^*–7 CO*], 841.4 (40) [M^+^*–8 CO*], 813.4 (57) [M^+^*–9 CO*], 785.4 (36) [M^+^*–10 CO*], 757.4 (40) [M^+^*–11 CO*], 729.5 (38) [M^+^*–12 CO*], 701.5 (45) [M^+^*–13 CO*], 673.5 (40) [M^+^*–14 CO*], 645.5 (46) [M^+^*–15 CO*], 617.6 (48) [M^+^*–16 CO*], 589.6 (33) [M^+^*–17 CO*], 561.5 (23) [M^+^*–18 CO*], 533.6 (100) [M^+^*–19 CO*]. Elemental analysis, calcd. for Co_8_P_2_C_19_O_19_ (1065.32 g/mol): C, 21.42. Found C, 21.30.

## 4. Conclusions

In the present study, we have demonstrated the high potential of [Co_2_(CO)_8_] to form cobalt clusters embedding P and As atoms in their core upon its reaction with various P and As sources, such as white phosphorus and yellow arsenic. Accordingly, a large number of novel, as well as reported, clusters were formed depending on the reaction conditions involved. Those include stoichiometry of the reactants, temperature, reaction time, method of crystallization, and solvent. The formed clusters are surrounded by carbonyl ligands. However, EI mass spectra reveal the successive loss of those CO ligands and the possibility of isolating the substituent-free metal-P or metal-As cluster cores. Our current efforts in this field focus on enlarging the family of these valuable candidates further and investigating their potential as single-source precursors for the synthesis of Co_x_P_y_ or Co_x_As_y_ nanoparticles with varied metal-to-main group element ratios.

## Data Availability

Data are contained within the article and Supplementary Materials.

## Supplementary Materials

The supplementary materials are available online at https://www.mdpi.com/article/10.3390/molecules29092025/s1. Figure S1. 1H NMR spectrum of **1**. Figure S2. 13C{1H} NMR spectrum of **1**. Figure S3. 31P{1H} NMR spectrum of **2**. Figure S4. 13C{1H} NMR spectrum of **2** (above) and **3** (below). Figure S5. 31P{1H} NMR spectrum of **4**. Figure S6. 13C{1H} NMR spectrum of **4**. Figure S7. 31P{1H} NMR spectrum of **6**. Figure S8. 31P{1H} NMR spectrum of **7**. Figure S9. 31P{1H} NMR spectrum of **8**. Figure S10. 31P{1H} MAS NMR spectrum of **9**. Figure S11. 31P{1H} NMR spectrum of **A**. Figure S12. Spin density distribution (left) and singly occupied natural orbital in [Co8(CO)16(μ-CO)4P] (**5**) in the doublet ground state, calculated on the r2SCAN-3c level (right). Figure S13. View of the asymmetric unit of **1.** Figure S14. View of the asymmetric unit of **2.** Figure S15. View of the asymmetric unit of **3.** Figure S16. View of the asymmetric unit of **4**. Figure S17. View of the asymmetric unit of **5.** Figure S18. View of the asymmetric unit of **6.** Figure S19. View of the asymmetric unit of **7**. Figure S20. View of the asymmetric unit of **8.** Figure S21. (a) View of the asymmetric unit of **9**; (b) Molecular structure of compound **9** in the solid state. Figure S22. (a) View of the asymmetric unit of **10**; (b) Molecular structure of compound **10** in the solid state. Figure S23. View of the asymmetric unit of **11.** Figure S24. View of the asymmetric unit of **12**. Table S1. Thermodynamic parameters (Hartree) calculated for **5** in different spin states at the r2SCAN-3c level of theory. Table S2. Crystallographic data for compounds **1**–**4**. Table S3. Crystallographic data for compounds **5**–**8**. Table S4. Crystallographic data for compounds **9**–**12**. References [29,31,37,38,39,40,41,42,43,44,45,46,47] are cited in the Supplementary Materials.
