# Supplementary material for: Synthesis and Characterization of Novel Cobalt Carbonyl Phosphorus and Arsenic Clusters
Source: Molecules. 2024 Apr 27;29(9):2025. doi: 10.3390/molecules29092025 (PMC11085420; doi:10.3390/molecules29092025)
Supplement: Supplementary file 1 [file molecules-29-02025-s001.zip › molecules-2961262-supplementary.pdf]

Supporting Information

Synthesis and characterization of novel cobalt carbonyl phosphorus and arsenic clusters

Mehdi Elsayed Moussa,† Susanne Bauer,† Christian Graßl, Christoph Riesinger, Gábor Balázs and Manfred Scheer[a]*

Department of Inorganic Chemistry, University of Regensburg, GER-93040 Regensburg (Germany)

† Authors contributed equally to this work

1. Synthesis and characterization of compound 1:

A solution of $W(CO)_6$ (2.5 g, 7.1 mmol) in THF (250 mL) was irradiated for five hours with a mercury vapor lamp. THF was removed under reduced pressure and the residue was solved in toluene (250 mL). To this solution $As(SiMe_3)_3$ (2.1 mL, 7.1 mmol) was added and the mixture was stirred for nine days at room temperature. Subsequently MeOH (1 mL, 26 mmol) was added and the solution was stirred for further three days (**2**) at room temperature. After removing the solvent under reduced pressure, crystals were obtained by sublimation of the residual solids at 45 °C at 10^{-3} mbar. Yield: 1.284 g (45 %). IR (KBr): $\tilde{\nu}/cm^{-1}$ = 2079 (m), 1990 (m), 1908 (br vs), 2196 (m), 903 (s), 577 (m). ^1H NMR (400.132 MHz, C_6D_6): δ[ppm] = 1.56 (s, 3 H, AsH_3). $^{13}C\{^1H\}$ NMR (100.613 MHz, C_6D_6): δ[ppm] = 195.7 (s, $^1J_{CW}$ = 125.6 Hz, CO), 198.1 (s, CO). EI-MS (70 eV): m/z (%) = 401.8 (83) [M^+], 373.8 (18) [M^+ – CO], 345.8 (52) [M^+ – 2 CO], 315.8 (96) [M^+ – 3 CO – 2 H], 286.8 (58) [M^+ – 4 CO – 3 H], 258.8 (35) [M^+ – 5 CO – 3 H]. Anal. Calcd for $CrC_5O_5AsH_3$ (269.86 g/mol): C, 22.24; H, 1.12. Found C, 22.80; H, 1.17 (**1**). Elemental analysis, calcd. for $WC_5O_5AsH_3$ (401.87 g/mol): C, 14.94; H, 0.75. Found C, 14.98; H, 0.63.

2. NMR spectra of compounds 1-4, 6-9 and A:

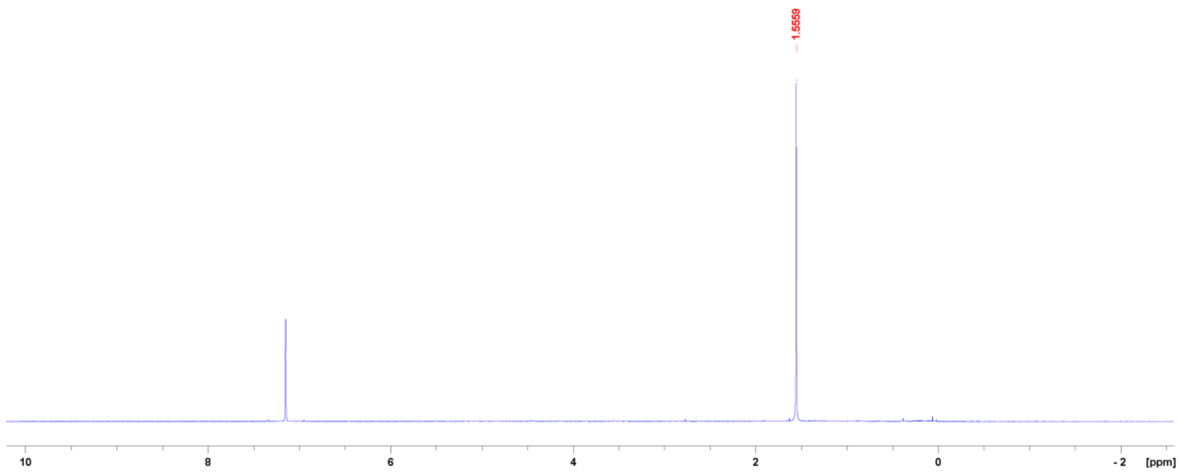

Figure S1. ^1H NMR spectrum of **1**.

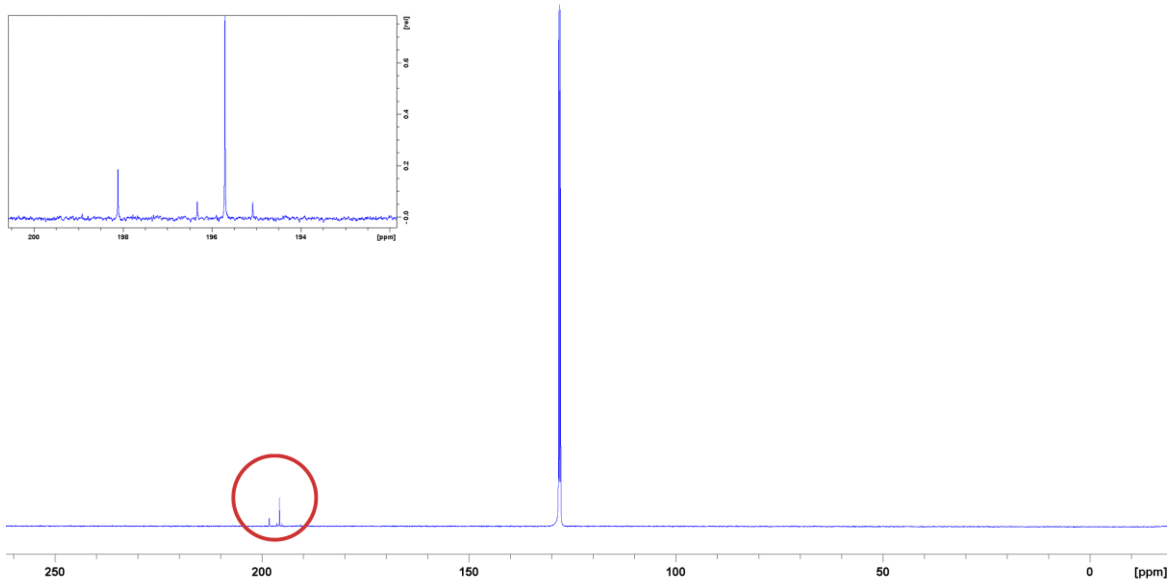

Figure S2. $^{13}C\{^1H\}$ NMR spectrum of **1**.

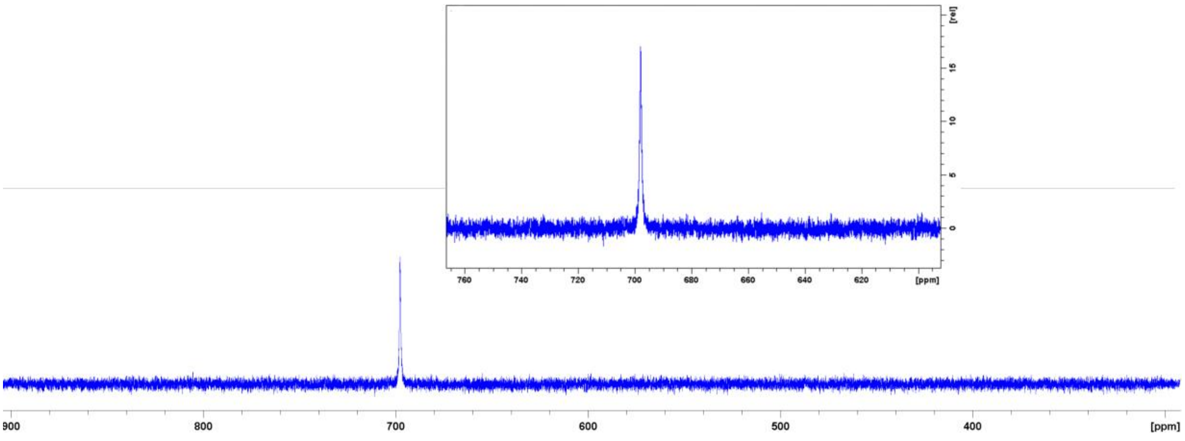

Figure S3. $^{31}P\{^{1}H\}$ NMR spectrum of **2**.

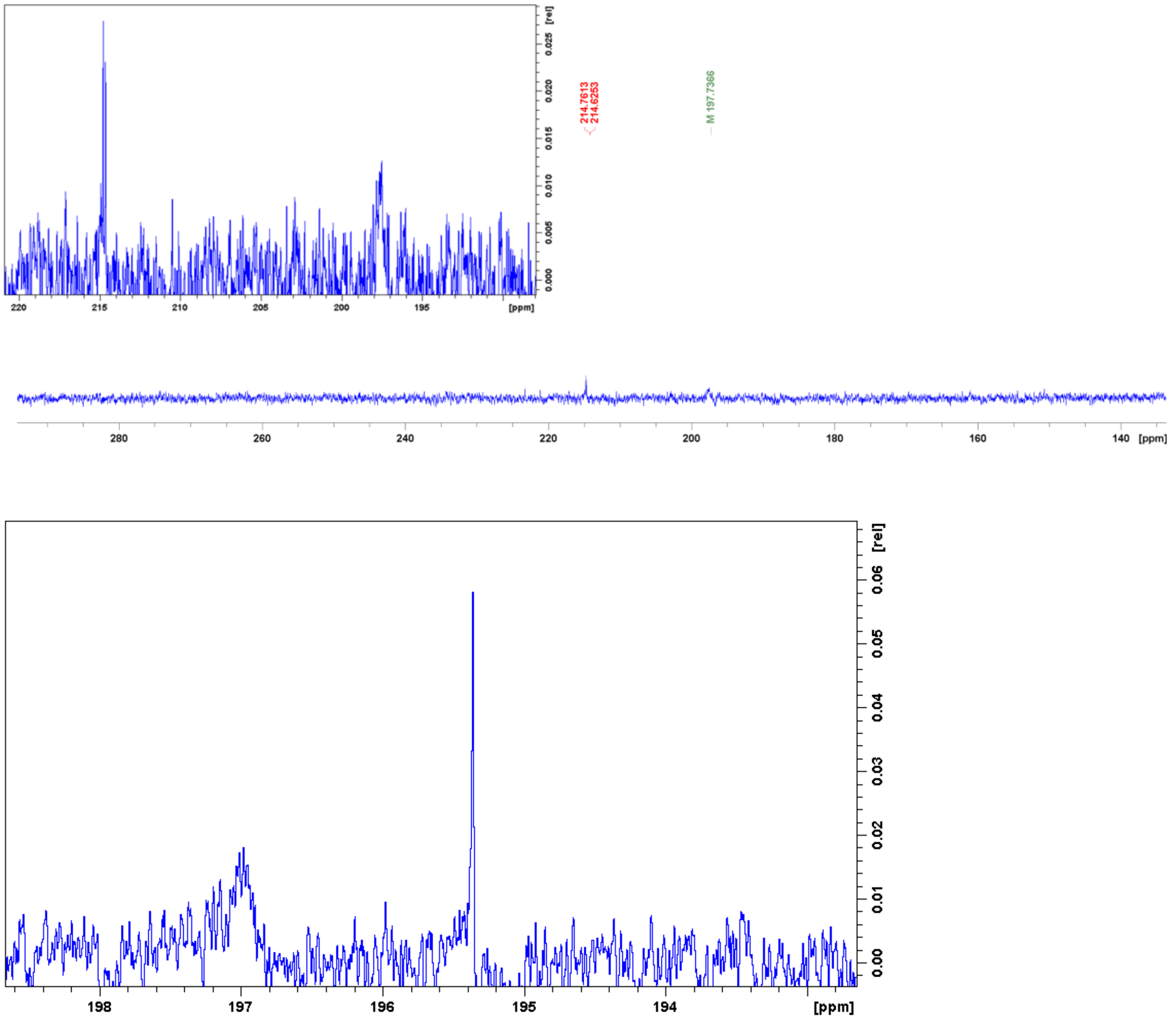

Figure S4. $^{13}C\{^{1}H\}$ NMR spectrum of **2** (above) and **3** (below).

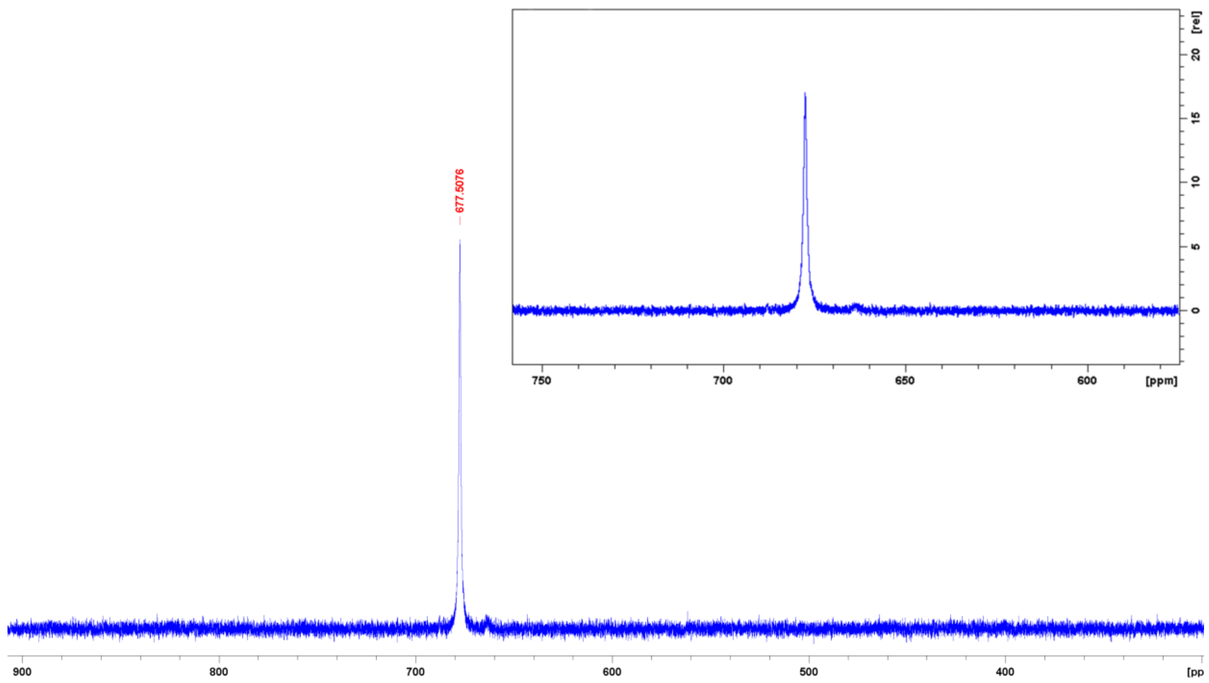

Figure S5. $^{31}P\{^{1}H\}$ NMR spectrum of **4**.

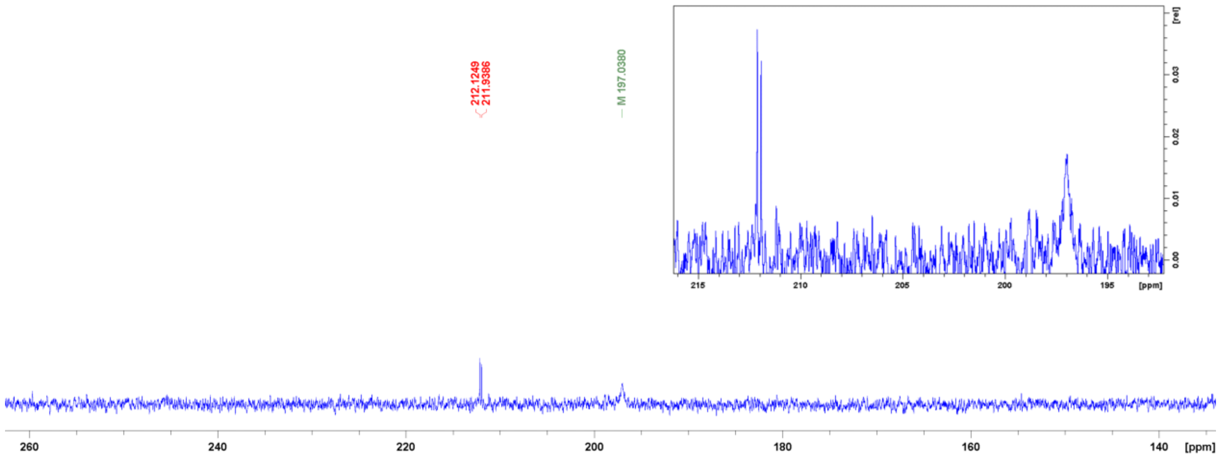

Figure S6. $^{13}C\{^{1}H\}$ NMR spectrum of **4**.

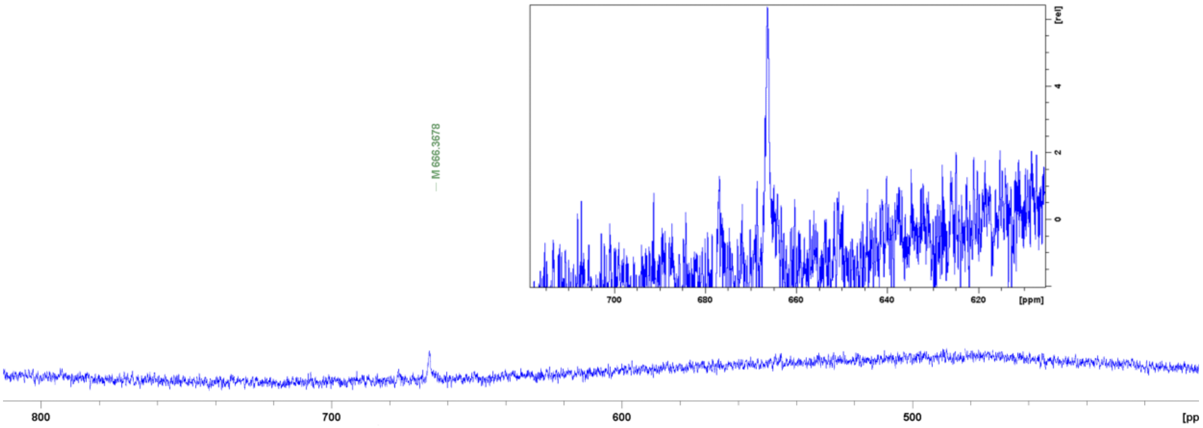

Figure S7. $^{31}P\{^1H\}$ NMR spectrum of **6**.

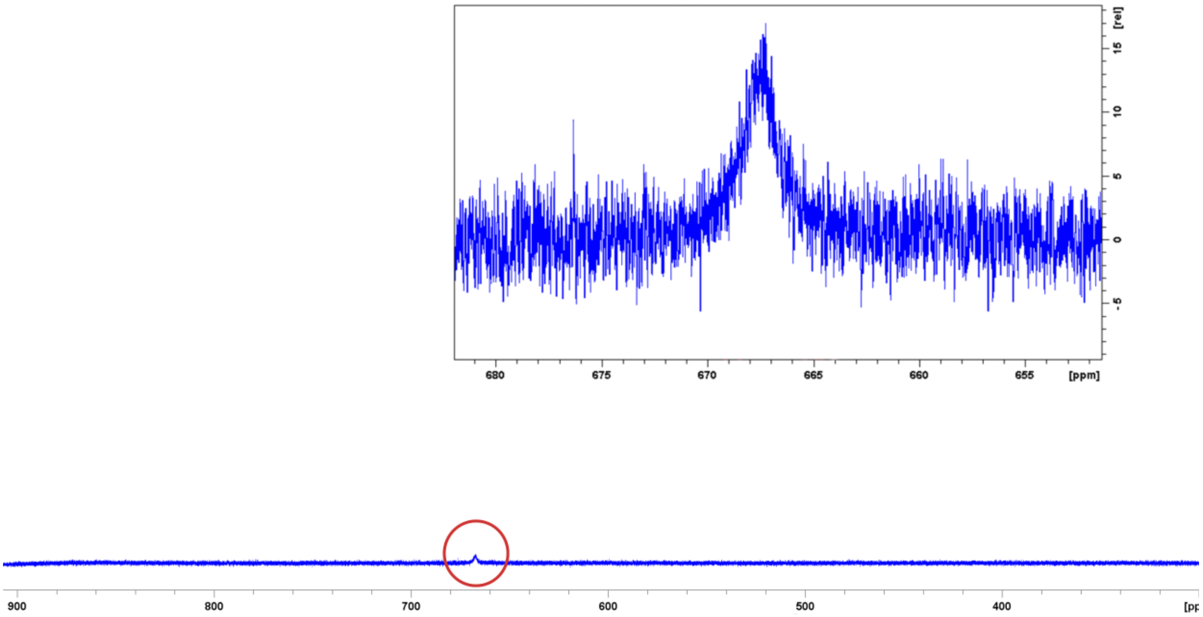

Figure S8. $^{31}P\{^1H\}$ NMR spectrum of **7**.

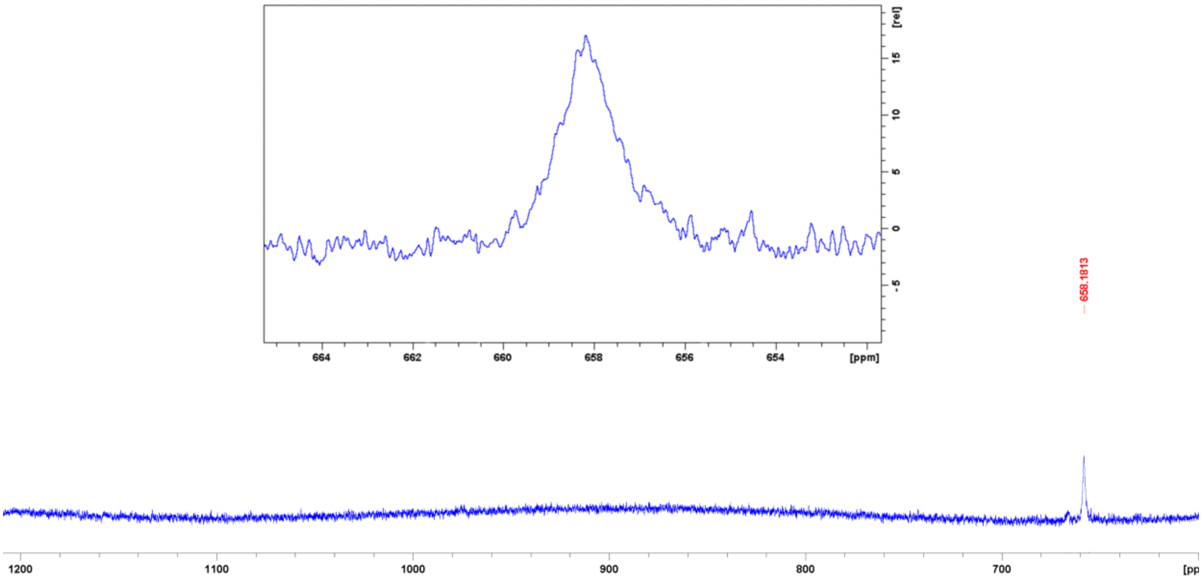

Figure S9. $^{31}P\{^1H\}$ NMR spectrum of **8**.

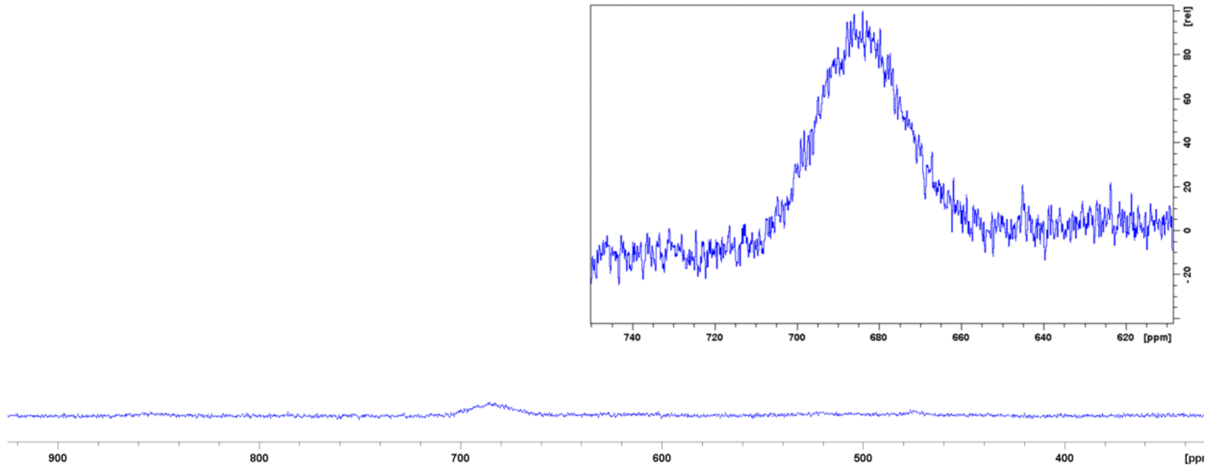

Figure S10. $^{31}P\{^{1}H\}$ MAS NMR spectrum of **9**.

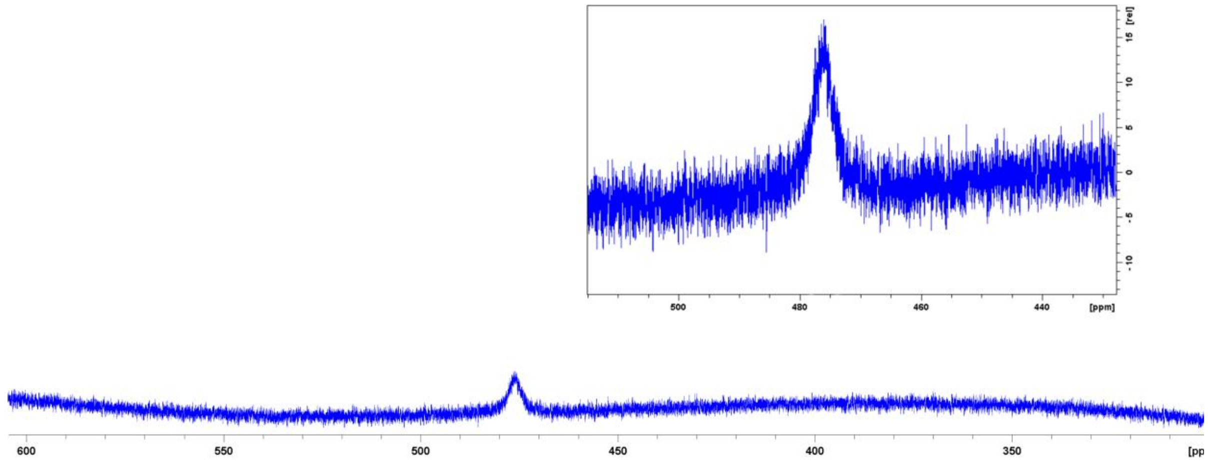

Figure S11. $^{31}P\{^{1}H\}$ NMR spectrum of **A**.

3. DFT calculations for cluster 5:

All calculations have been performed using ORCA program, version 5.0.3.[1] For the geometry optimization and frequency analysis the r^{2}SCAN-3c[2] composite method has been used, which includes geometrical counterpoise correction gCP[3] and the atom-pairwise dispersion correction based on tight binding partial charges (D4)[4] as well as the def2-mTZVPP auxiliary and orbital basis set.[2]

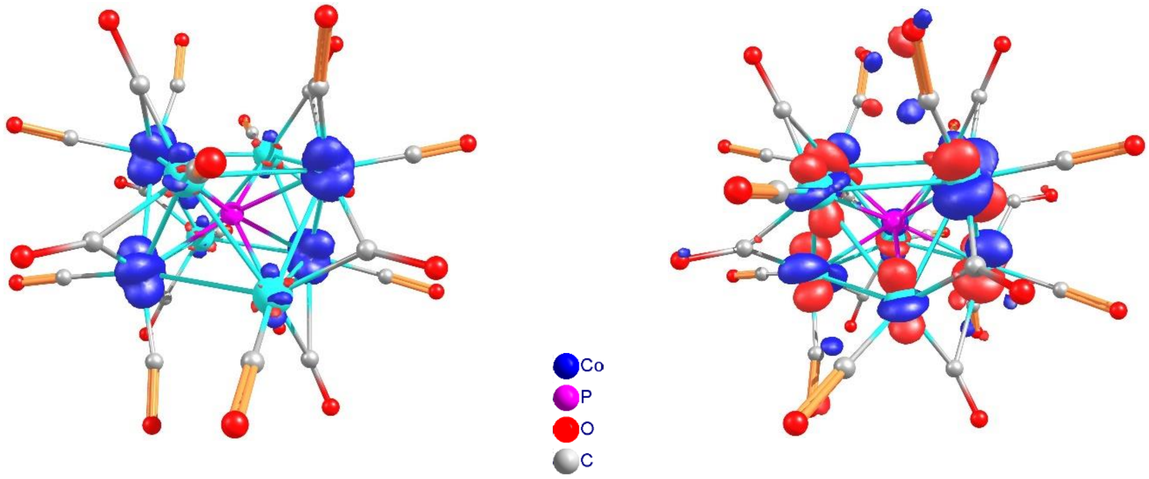

Figure S12. Spin density distribution (left) and singly occupied natural orbital in $[Co_8(CO)_{16}(\mu\text{-}CO)_4P]$ (**5**) in the doublet ground state, calculated on the r^2SCAN-3c level (right).

Table S1. Thermodynamic parameters (Hartree) calculated for **5** in different spin states at the r^2SCAN-3c level of theory.

	Doublet	Quartet
Electronic energy	-13670.85694	-13670.83486
Total thermal energy	-13670.6203	-13670.59948
Total Enthalpy	-13670.61936	-13670.59854
Final entropy term	0.14625567	0.14938362
Final Gibbs free energy	-13670.76561	-13670.74792

Cartesian coordinates of the optimized geometry of **5** in the doublet ground state.

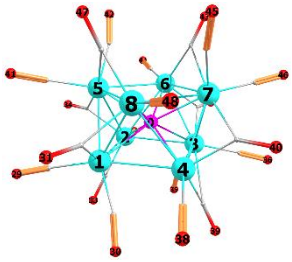

P	-0.00689087811709	0.01781563073716	-0.00698516371747
Co	1.47113072242262	-0.43231674132434	-1.63228177612458
Co	-0.88926142695194	-1.65693061002599	-1.18112330031444
Co	-2.04354991986912	0.81340952242140	-0.50419627120172
Co	0.33239944486580	2.03673055781503	-0.87996841827351
Co	1.06275230017859	-1.86948141566769	0.55904409895613
Co	-1.48254723317514	-0.96346995104361	1.34162431822358
Co	-0.52821459249870	1.54788978276751	1.55107255713748
Co	2.00809202547704	0.65292840929022	0.69452753892558
C	2.38707261774865	-1.74120272228376	-2.41763876558329
C	1.35974291508269	0.69890165236333	-3.01073921231347
C	3.10525653974935	0.22376085521130	-0.79801601821159
C	-2.36858023848004	-2.62568170782218	-1.31938420774495
C	-0.37878719322265	-1.63450563294314	-2.88650373880650
C	0.11602581915524	-3.14986640534624	-0.56301231704483
C	-2.68113580776127	0.15134419356700	-2.03642862762186
C	-3.13005103578248	2.21379013978549	-0.33854722381433
C	-3.12408323288351	-0.19293963097518	0.76407652146175
C	1.63900219974243	3.10823090338180	-1.41724385113345
C	-0.92759221705627	2.48270621251858	-2.05516933441776
C	-0.12830996360704	3.18330107858380	0.55977740836028
C	2.56811958145972	-2.79858260124303	0.36434906194400
C	0.57969098024872	-2.45835322258430	2.17591729404360
C	-1.40244677088192	-0.30418616008550	2.99435990541901

C	-2.25618412978075	-2.52374639035135	1.67735371690579
C	0.70174889957957	1.68017026583582	2.84053579869464
C	-1.88146734814378	2.37276876534467	2.36239174965479
C	2.68468300134903	-0.46484820038171	1.90313776153444
C	2.95689218248607	2.10250467598814	1.07391991701454
O	2.97948342438390	-2.55668553314067	-2.95651065583532
O	1.40752256520394	1.25804325301420	-4.01260965760509
O	4.23103032529364	0.30577138501735	-1.09412806368816
O	-3.31797274968987	-3.25267948116315	-1.43801392145898
O	-0.31079915245669	-1.81436186165607	-4.02289984528175
O	0.16013571626891	-4.30006167976263	-0.75499536658423
O	-3.24559584860880	-0.07426264106704	-3.01118909927878
O	-3.84977166695981	3.09867545707062	-0.26685710295740
O	-4.25713257896892	-0.27249758404781	1.03192190549094
O	2.46578149510963	3.80747142643694	-1.78618386015016
O	-1.51240949882414	2.98721538301479	-2.91068710839341
O	-0.16377697953223	4.32879630598297	0.77950182367110
O	3.52520145713105	-3.41683423863225	0.27469931006444
O	0.48751737672642	-2.96915742171267	3.20085886298878
O	-1.51766541064883	-0.17767726628737	4.13479127865943
O	-2.75635617151135	-3.52307101121780	1.92112158030324
O	1.31646814818605	1.87397120251329	3.79096555647388
O	-2.72355840058364	2.91300418656399	2.91491975285454
O	3.31740728725749	-0.92499909584859	2.74974695168417
O	3.58148342088945	3.02199796138863	1.34359823709091

MULLIKEN ATOMIC CHARGES AND SPIN POPULATIONS

0	P :	0.478188	-0.069218
1	Co:	-0.541429	0.251200
2	Co:	-0.540763	0.020036
3	Co:	-0.541199	0.251499
4	Co:	-0.541762	0.025440
5	Co:	-0.541697	0.244299
6	Co:	-0.540411	0.022489
7	Co:	-0.548325	0.246757
8	Co:	-0.539466	0.030281

Mayer bond orders larger than 0.100000

B(0-P , 1-Co) :	0.4147	B(0-P , 2-Co) :	0.4267	B(0-P , 3-Co) :	0.4132
B(0-P , 4-Co) :	0.4290	B(0-P , 5-Co) :	0.4141	B(0-P , 6-Co) :	0.4255
B(0-P , 7-Co) :	0.4131	B(0-P , 8-Co) :	0.4272	B(0-P , 11-C) :	-0.1006
B(0-P , 20-C) :	-0.1001	B(1-Co, 2-Co) :	0.3255	B(1-Co, 4-Co) :	0.2587
B(1-Co, 5-Co) :	0.3241	B(1-Co, 8-Co) :	0.3174	B(1-Co, 9-C) :	1.1555
B(1-Co, 10-C) :	1.1796	B(1-Co, 11-C) :	0.8049	B(1-Co, 13-C) :	0.2444
B(1-Co, 29-O) :	0.1439	B(1-Co, 30-O) :	0.1574	B(1-Co, 31-O) :	0.1234
B(2-Co, 3-Co) :	0.2623	B(2-Co, 5-Co) :	0.3151	B(2-Co, 6-Co) :	0.2366
B(2-Co, 12-C) :	1.1847	B(2-Co, 13-C) :	1.1779	B(2-Co, 14-C) :	0.8742
B(2-Co, 15-C) :	0.1722	B(2-Co, 32-O) :	0.1516	B(2-Co, 33-O) :	0.1631
B(2-Co, 34-O) :	0.1448	B(3-Co, 4-Co) :	0.3239	B(3-Co, 6-Co) :	0.3157
B(3-Co, 7-Co) :	0.3234	B(3-Co, 15-C) :	1.1765	B(3-Co, 16-C) :	1.1560
B(3-Co, 17-C) :	0.8043	B(3-Co, 19-C) :	0.2441	B(3-Co, 35-O) :	0.1578
B(3-Co, 36-O) :	0.1442	B(3-Co, 37-O) :	0.1242	B(4-Co, 7-Co) :	0.3163
B(4-Co, 8-Co) :	0.2382	B(4-Co, 10-C) :	0.1603	B(4-Co, 18-C) :	1.1871
B(4-Co, 19-C) :	1.1795	B(4-Co, 20-C) :	0.8840	B(4-Co, 38-O) :	0.1514
B(4-Co, 39-O) :	0.1627	B(4-Co, 40-O) :	0.1458	B(5-Co, 6-Co) :	0.2605
B(5-Co, 8-Co) :	0.3255	B(5-Co, 14-C) :	0.8033	B(5-Co, 21-C) :	1.1568
B(5-Co, 22-C) :	1.1747	B(5-Co, 27-C) :	0.2479	B(5-Co, 34-O) :	0.1238
B(5-Co, 41-O) :	0.1441	B(5-Co, 42-O) :	0.1577	B(6-Co, 7-Co) :	0.3273
B(6-Co, 17-C) :	0.8735	B(6-Co, 22-C) :	0.1733	B(6-Co, 23-C) :	1.1718
B(6-Co, 24-C) :	1.1864	B(6-Co, 37-O) :	0.1448	B(6-Co, 43-O) :	0.1636
B(6-Co, 44-O) :	0.1518	B(7-Co, 8-Co) :	0.2588	B(7-Co, 20-C) :	0.7971
B(7-Co, 23-C) :	0.2618	B(7-Co, 25-C) :	1.1781	B(7-Co, 26-C) :	1.1551
B(7-Co, 40-O) :	0.1223	B(7-Co, 45-O) :	0.1580	B(7-Co, 46-O) :	0.1439
B(8-Co, 11-C) :	0.8770	B(8-Co, 25-C) :	0.1618	B(8-Co, 27-C) :	1.1792
B(8-Co, 28-C) :	1.1865	B(8-Co, 31-O) :	0.1453	B(8-Co, 47-O) :	0.1632
B(8-Co, 48-O) :	0.1516	B(9-C , 29-O) :	2.1426	B(10-C , 30-O) :	2.0708
B(11-C , 31-O) :	1.9661	B(12-C , 32-O) :	2.1317	B(13-C , 33-O) :	2.0366

```
B( 14-C , 34-O ) :   1.9665 B( 15-C , 35-O ) :   2.0672 B( 16-C , 36-O ) :   2.1429
B( 17-C , 37-O ) :   1.9659 B( 18-C , 38-O ) :   2.1308 B( 19-C , 39-O ) :   2.0363
B( 20-C , 40-O ) :   1.9665 B( 21-C , 41-O ) :   2.1433 B( 22-C , 42-O ) :   2.0665
B( 23-C , 43-O ) :   2.0325 B( 24-C , 44-O ) :   2.1324 B( 25-C , 45-O ) :   2.0705
B( 26-C , 46-O ) :   2.1434 B( 27-C , 47-O ) :   2.0354 B( 28-C , 48-O ) :   2.1309
```

```
--------------------------
THERMOCHEMISTRY AT 298.15K
--------------------------
Temperature          ... 298.15 K
Pressure             ... 1.00 atm
Total Mass           ... 1062.61 AMU

INNER ENERGY

Summary of contributions to the inner energy U:
Electronic energy                ... -13670.85693865 Eh
Zero point energy                ...      0.17693682 Eh     111.03 kcal/mol
Thermal vibrational correction   ...      0.05686998 Eh      35.69 kcal/mol
Thermal rotational correction    ...      0.00141627 Eh       0.89 kcal/mol
Thermal translational correction ...      0.00141627 Eh       0.89 kcal/mol
-----------------------------------------------------------------------
Total thermal energy                 -13670.62029931 Eh

ENTHALPY

Total free energy                  ... -13670.62029931 Eh
Thermal Enthalpy correction        ...      0.00094421 Eh       0.59 kcal/mol
-----------------------------------------------------------------------
Total Enthalpy                     ... -13670.61935510 Eh

ENTROPY

Electronic entropy                 ...      0.00065446 Eh       0.41 kcal/mol
Vibrational entropy                ...      0.10513140 Eh      65.97 kcal/mol
Rotational entropy                 ...      0.01825147 Eh      11.45 kcal/mol
Translational entropy              ...      0.02221834 Eh      13.94 kcal/mol
-----------------------------------------------------------------------
Final entropy term                 ...      0.14625567 Eh      91.78 kcal/mol

GIBBS FREE ENERGY

Total enthalpy                     ... -13670.61935510 Eh
Total entropy correction           ...     -0.14625567 Eh     -91.78 kcal/mol
-----------------------------------------------------------------------
Final Gibbs free energy          ... -13670.76561076 Eh
```

Cartesian coordinates of the optimized geometry of **5** in the quartet spin state.

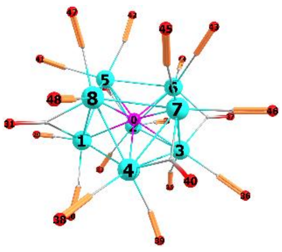

```
P   -0.01088828652097    0.01487772096523   -0.00439339725513
Co   1.50836707368441   -0.34468015116940   -1.61668435086094
Co  -0.93625996832271   -1.66888445672635   -1.16803025383301
Co  -2.08299122303946    0.71800898728679   -0.50781784094415
Co   0.38006832335092    2.03575795591448   -0.90220840526504
Co   1.02671866236049   -1.89999122868067    0.53942971606799
Co  -1.49219454621930   -0.94828791466793    1.37662136885604
Co  -0.51198424870604    1.59236063655493    1.51510503783517
Co   2.02260484366037    0.63427164657034    0.71981650130556
C    2.34645819933881   -1.63961656746772   -2.49345722874278
C    1.33242569708315    0.77353835202046   -2.99267950943161
C    3.16466152886216    0.25016000822043   -0.80048336523836
C   -2.43021990322130   -2.64920753377472   -1.19092741966393
C   -0.52093817866093   -1.75525257779849   -2.91595825308129
C    0.09027716132170   -3.17277603555279   -0.55435384097135
C   -2.67463285339409    0.08185630980471   -2.06307842105850
C   -3.12777209860190    2.15091972990782   -0.44721965219611
C   -3.18719290058414   -0.21862098036428    0.77943986045074
C    1.76299867980618    3.07887187754803   -1.33931019050417
C   -0.80707231441765    2.62285381268548   -2.11801078815083
C   -0.10077292998926    3.20088743359726    0.54726918627475
```

C	2.58111642194405	-2.73111239375985	0.24765693395046
C	0.63573991633590	-2.59413018500358	2.15161270725677
C	-1.37633633537279	-0.34158989350842	3.04706626432307
C	-2.24111883030213	-2.50984472403364	1.76218228783188
C	0.65552150841808	1.79820490164796	2.86776042305221
C	-1.94100625102041	2.36180046269502	2.26305652776652
C	2.66973707686673	-0.45112263649343	1.97520718508196
C	2.98522104123776	2.06054611771862	1.15246008573731
O	2.89240026636978	-2.44965765731425	-3.08827488594085
O	1.36006282166497	1.30975590921814	-4.01071680970971
O	4.29172162118395	0.33535669657086	-1.08375317181134
O	-3.37987988719456	-3.28215582216534	-1.26191778447938
O	-0.41510926483138	-1.91840945733565	-4.04680553815937
O	0.12855453059140	-4.32196402256211	-0.75352112455972
O	-3.23031427795878	-0.12703365420286	-3.04911015753385
O	-3.82112940834979	3.06045598754975	-0.43844919812000
O	-4.31539836316980	-0.30304026993166	1.05881192157780
O	2.61796260963576	3.76602492804834	-1.66260638736898
O	-1.42477809685000	3.11933159671403	-2.94770921681343
O	-0.12908389805254	4.35008868814633	0.74837647831240
O	3.57243641841686	-3.28496240393088	0.11285645344948
O	0.54891790361526	-3.13565680348919	3.15941485519933
O	-1.43466735235083	-0.17862809844158	4.18508611266037
O	-2.73057690784071	-3.50395233702987	2.04559645672563
O	1.25486484789241	2.02028824734794	3.82060027496215
O	-2.82272884500669	2.86391322224261	2.79024567017907
O	3.24855575198373	-0.94345754714429	2.83971312871019
O	3.62815426435334	2.95670412357337	1.45499175412697

4. Crystallographic details:

The crystallographic data for all described compounds were collected on an Xcalibur, Ruby, Gemini Ultra (Rigaku) with an AtlasS2 detector using Cu–Kα (**3**,) or Mo–Kα (**1**, **6**, **7**, **9**, **10**, **11**, **12**) radiation or an Agilent SuperNova diffractometer with Cu–Kα (**5**, **8**) or Mo–Kα (**2**, **4**) radiation. Data reduction and absorption correction were performed with the CrysAlisPro software package.[5] Structure solution and refinement was conducted in Olex2 (1.5 alpha)[6] with ShelXT[7] (solution) and ShelXL 2018/3[8] (least squares refinement (F2)). All non H atoms were refined with anisotropic displacement parameters and H atoms were treated as riding models with isotropic displacement parameters and fixed C–H bond lengths (sp3: 0.96 (CH3), 0.97 (CH2); sp2: 0.93 (CH)). Visualization of the crystal structures was performed with Olex2 (1.5-alpha).[6]

Compound 1: Compound **1** crystallizes from sublimation as colorless plate-shaped crystals with the monoclinic space group $P2_1/c$. The asymmetric unit contains one molecule of **1**.

Compound 2: Compound **2** crystallizes from concentrated *n*-hexane solutions at -25 °C as dark red block-shaped crystals with the monoclinic space group *Cc*. The asymmetric unit contains one molecule of **2**.

Compound 3: Compound **3** crystallizes from concentrated *n*-hexane solutions at -25 °C as dark violet block-shaped crystals with the triclinic space group $P\bar{1}$. The asymmetric unit contains one molecule of **3**.

Compound 4: Compound **4** crystallizes from concentrated *n*-hexane solutions at -25 °C as black needle-shaped crystals with the monoclinic space group $P2_1/n$. The asymmetric unit contains one molecule of **4**.

Compound 5: Compound **5** crystallizes from concentrated CH_2Cl_2 solutions at -25 °C as black plate-shaped crystals with the monoclinic space group $P2_1/n$. The asymmetric unit contains one molecule of **5**.

Compound 6: Compound **6** crystallizes from concentrated toluene solutions at -25 °C as black plate-shaped crystals with the triclinic space group $P\bar{1}$. The asymmetric unit contains one molecule of **6**.

Compound 7: Compound **7** crystallizes from concentrated *n*-hexane solutions at 8 °C as black block-shaped crystals with the monoclinic space group $P2_1/n$. The asymmetric unit contains one molecule of **7**. Disorder of two of the CO ligands was treated with appropriate restraints.

Compound 8: Compound **8** crystallizes from concentrated *n*-hexane solutions at -25 °C as black needle-shaped crystals with the orthorhombic space group $P2_12_12_1$. The asymmetric unit contains one molecule of **8**. However, poor crystal quality did only allow for collection of an incomplete data set, proving the structure of **8**, but not allowing structural analysis in detail.

Compound 9: Compound **9** crystallizes from concentrated *n*-hexane/toluene mixtures at room temperature as black needle-shaped crystals with the monoclinic space group $P2_1/c$. The asymmetric unit contains half of a molecule of **9**.

Compound 10: Compound **10** crystallizes from concentrated toluene solutions at -25 °C as black block-shaped crystals with the monoclinic space group $C2/c$. The asymmetric unit contains half of a molecule of **10** as well as one toluene molecule.

Compound 11: Compound **11** crystallizes from concentrated toluene solutions at -25 °C as black block-shaped crystals with the monoclinic space group $P2_1/n$. The asymmetric unit contains one molecule of **11**. Notably, another polymorph of **11** has already been described in the literature.[9]

Compound 12: Compound **12** crystallizes from concentrated *n*-hexane solutions at -25 °C as black rod-shaped crystals with the monoclinic space group $P2_1/n$. The asymmetric unit contains one molecule of **12**. Notably, another polymorph of **12** has already been described in the literature.[10]

CCDC-2343202 (**1**), CCDC-2343203 (**2**), CCDC-2343204 (**3**), CCDC-2343205 (**4**), CCDC-2343206 (**5**), CCDC-2343207 (**6**), CCDC-2343208 (**7**), CCDC-2343817 (**8**), CCDC-2343209 (**9**), CCDC-2343210 (**10**), CCDC-2343211 (**11**) and CCDC-2343212 (**12**) contain the supplementary crystallographic data for this paper. These data can be obtained free of charge at www.ccdc.cam.ac.uk/conts/retrieving.html (or from the Cambridge Crystallographic Data Centre, 12 Union Road, Cambridge CB2 1EZ, UK; Fax: + 44-1223-336-033; email: deposit@ccdc.cam.ac.uk).

Table S2. Crystallographic data for compounds **1**-**4**.

**Compound**	**1**	**2**	**3**	**4**
CCDC-number	2343202	2343203	2343204	2343205
Formula	$C_5H_3O_5AsW$	$C_{14}Co_3CrO_{14}P$	$AsC_{14}Co_3O_{14}W$	$C_{13}Co_3FeO_{13}P$
$D_{calc.}$ / g · cm^{-3}	2.938	2.066	2.578	2.099
μ/mm^{-1}	16.307	23.837	30.063	26.413
Formula Weight	401.84	651.90	827.70	627.74
Colour	colourless	dark red	black	black
Shape	block-shaped	block-shaped	block-shaped	needle-shaped
Size/mm^3	0.24×0.11×0.05	0.38×0.24×0.01	0.27×0.20×0.13	0.10×0.05×0.02
T/K	123.00(14)	123(1)	123.00(14)	123(1)
Crystal System	monoclinic	monoclinic	triclinic	monoclinic
Space Group	$P2_1/c$	Cc	$P\bar{1}$	$P2_1/n$
a/Å	10.8246(9)	15.2345(6)	8.7183(4)	8.7530(8)
b/Å	6.7417(4)	8.8120(2)	9.0608(4)	15.645(2)
c/Å	12.5789(8)	17.3130(8)	14.8480(6)	14.5092(18)
α/°	90	90	73.230(4)	90
β/°	98.184(7)	115.611(4)	83.682(3)	90.447(9)

γ/°	90	90	71.781(4)	90
V/Å^3	908.61(11)	2095.85(15)	1066.47(8)	1986.9(4)
Z	4	4	2	4
Z'	1	1	1	1
Wavelength/Å	0.71073	1.54178	1.54178	1.54178
Radiation type	MoK_α	CuK_α	CuK_α	CuK_α
θ_{min}/°	3.437	5.668	3.109	4.155
θ_{max}/°	29.349	76.435	66.554	72.995
Measured Refl.	3854	7526	13064	7542
Independent Refl.	2109	3528	3737	3758
Reflections with I > 2(I)	1521	3460	3647	2631
R_{int}	0.0611	0.0405	0.0449	0.0518
Parameters	110	299	298	280
Restraints	9	8	0	0
Largest Peak	2.337	1.159	3.112	0.773
Deepest Hole	-3.081	-0.853	-2.293	-0.666
GooF	1.057	1.085	1.072	1.017
wR_2 (all data)	0.1336	0.1565	0.0963	0.1324
wR_2	0.1157	0.1558	0.0951	0.1179
R_1 (all data)	0.0837	0.0574	0.0364	0.0779
R_1	0.0563	*Cc*	0.0356	0.0483
Flack Parameter	/	0.114(10)	/	/

Table S3. Crystallographic data for compounds **5**-**8**.

**Compound**	**5**	**6**	**7**	**8**
CCDC-number	2343206	2343207	2343208	2343817
Formula	$C_{20}Co_8O_{20}P$	$C_{20}Co_7O_{20}P$	$C_{24}Co_9O_{24}P_3$	$C_{21}Co_9O_{21}P_3$
$D_{calc.}$ / g · cm^{-3}	2.413	2.158	2.216	2.314
μ/mm^{-1}	35.960	3.801	3.953	34.790
Formula Weight	1062.61	1003.68	1295.52	1211.49
Colour	black	black	black	black
Shape	plate-shaped	plate-shaped	block-shaped	needle-shaped
Size/mm^3	0.15×0.07×0.02	0.19×0.10×0.04	0.37×0.09×0.05	0.13×0.04×0.02
T/K	123(1)	123.1(6)	123.00(14)	123(1)
Crystal System	monoclinic	triclinic	monoclinic	orthorhombic
Space Group	$P2_1/n$	$P\bar{1}$	$P2_1/n$	$P2_12_12_1$
a/Å	9.8425(3)	8.6584(4)	9.4707(3)	9.8379(8)
b/Å	17.5260(6)	11.0824(4)	18.8780(6)	12.6588(6)
c/Å	16.9685(5)	17.4356(5)	21.9690(6)	27.9292(18)
α/°	90	91.480(3)	90	90
β/°	92.151(3)	96.904(3)	98.605(2)	90
γ/°	90	111.126(4)	90	90
V/Å^3	2925.00(16)	1544.96(11)	3883.6(2)	3478.2(4)
Z	4	2	4	4
Z'	1	1	1	1
Wavelength/Å	1.54178	0.71073	0.71073	1.54178
Radiation type	CuK_α	MoK_α	MoK_α	CuK_α
θ_{min}/°	3.627	2.963	2.862	4.715

θ_{max}/°	73.100	29.489	29.507	66.482
Measured Refl.	9758	40289	39370	5650
Independent Refl.	5602	7792	9514	3964
Reflections with I > 2(I)	4396	6325	8458	3451
R_{int}	0.0629	0.0486	0.0337	0.0459
Parameters	442	433	577	487
Restraints	0	0	54	24
Largest Peak	0.989	1.193	0.838	1.213
Deepest Hole	-1.056	-0.649	-0.632	-1.432
GooF	1.036	1.044	1.270	1.013
wR_2 (all data)	0.1740	0.0953	0.0965	0.1663
wR_2	0.1585	0.0886	0.0940	0.1574
R_1 (all data)	0.0808	0.0571	0.0515	0.0688
R_1	0.0641	0.0407	0.0439	0.0609
Flack Parameter	/	/	/	-0.026(11)

Table S4. Crystallographic data for compounds **9**-**12**.

**Compound**	**9**	**10**	**11**	**12**
CCDC-number	2343209	2343210	2343211	2343212
Formula	$C_{26}Co_{10}O_{26}P_4$	$C_{44}H_{16}Co_{16}O_{30}P_6$	$As_3C_{24}Co_9O_{24}$	$AsC_{20}Co_7O_{20}$
$D_{calc.}$ / g · cm^{-3}	2.267	2.177	2.436	2.271
μ/mm^{-1}	4.061	4.147	6.351	4.853
Formula Weight	1441.44	2153.27	1427.37	1047.63
Colour	black	black	black	black
Shape	needle-shaped	cube-shaped	block-shaped	plate-shaped
Size/mm^3	0.22×0.14×0.01	0.11×0.10×0.06	0.86×0.52×0.45	0.50×0.16×0.05
T/K	123.00(14)	122.9(5)	123.1(3)	122.95(10)
Crystal System	monoclinic	monoclinic	monoclinic	monoclinic
Space Group	$P2_1/c$	$C2/c$	$P2_1/n$	$P2_1/n$
a/Å	11.9865(5)	27.660(3)	11.2417(4)	8.7211(3)
b/Å	10.2655(7)	12.8188(10)	21.1186(6)	31.9682(11)
c/Å	17.7539(12)	21.965(3)	16.6478(5)	11.2058(4)
α/°	90	90	90	90
β/°	104.824(5)	122.486(16)	99.977(3)	101.294(4)
γ/°	90	90	90	90
V/Å^3	2111.9(2)	6569.6(15)	3892.6(2)	3063.65(19)
Z	2	4	4	4
Z'	1	0.5	1	1
Wavelength/Å	0.71073	0.71073	0.71073	0.71073
Radiation type	MoK_α	MoK_α	MoK_α	MoK_α
θ_{min}/°	3.083	3.064	3.430	3.489
θ_{max}/°	29.432	29.271	29.555	29.567
Measured Refl.	17258	19008	34708	12863

Independent Refl.	5151	7615	9816	6965
Reflections with I > 2(I)	4194	5971	7529	6084
R_{int}	0.0406	0.0355	0.0795	0.0296
Parameters	298	423	541	433
Restraints	0	0	0	0
Largest Peak	0.550	1.820	1.277	1.331
Deepest Hole	-0.475	-0.500	-1.163	-0.683
GooF	1.045	1.017	1.015	1.069
wR_2 (all data)	0.0681	0.0912	0.0976	0.0797
wR_2	0.0618	0.0844	0.0948	0.0757
R_1 (all data)	0.0468	0.0571	0.0557	0.0393
R_1	0.0321	0.0385	0.0418	0.0320
Flack Parameter	/	/	/	/

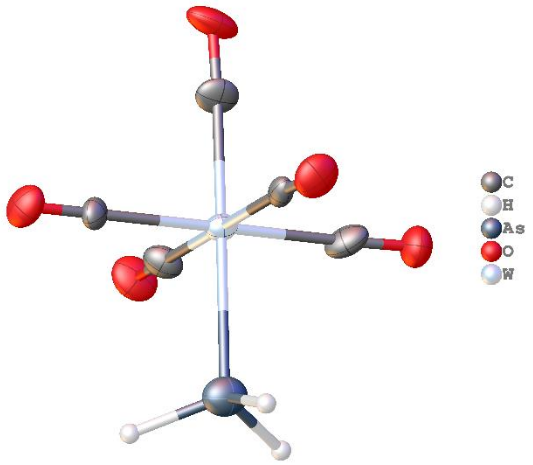

Fig. S13. View of the asymmetric unit of **1**.

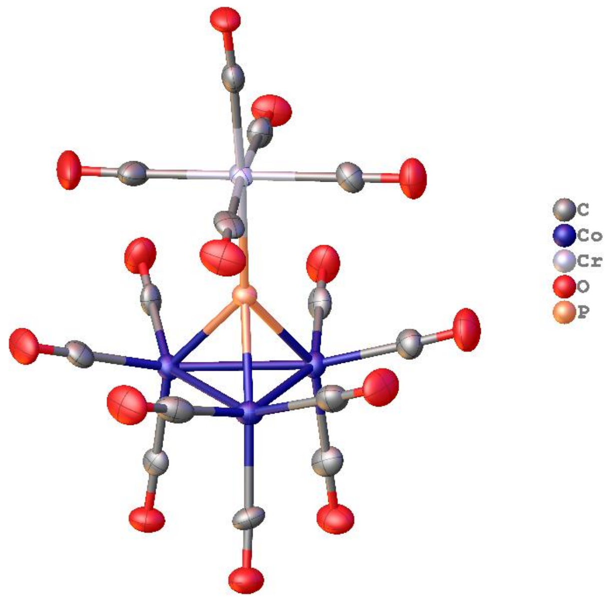

Fig. S14. View of the asymmetric unit of **2**.

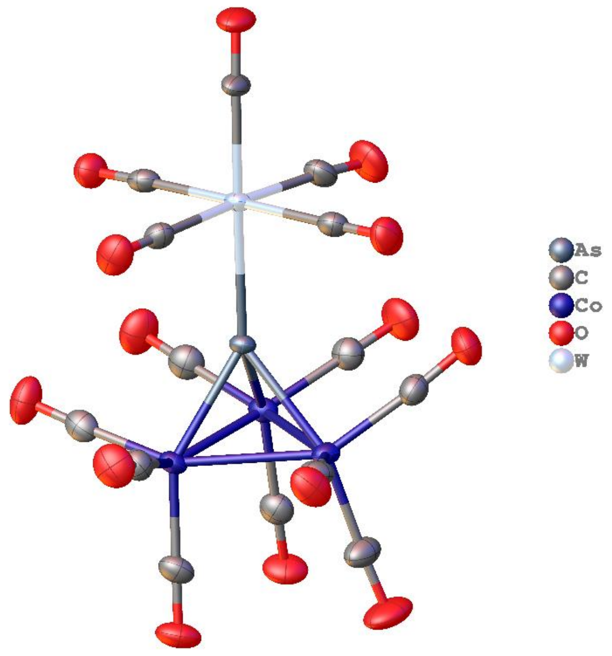

Fig. S15. View of the asymmetric unit of **3**.

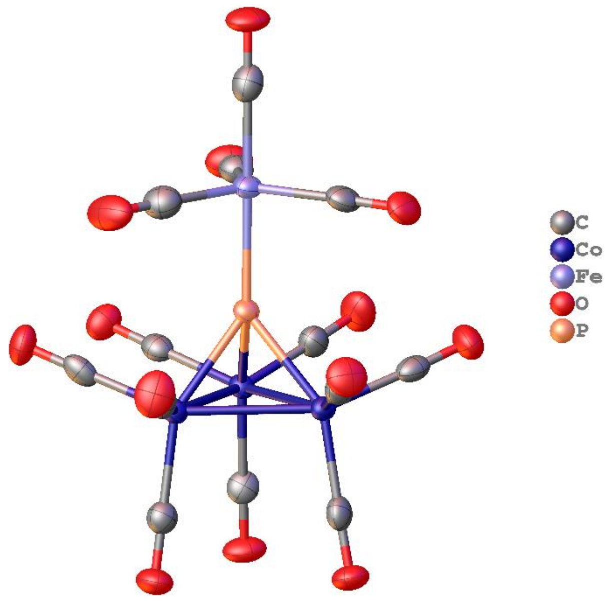

Fig. S16. View of the asymmetric unit of **4**.

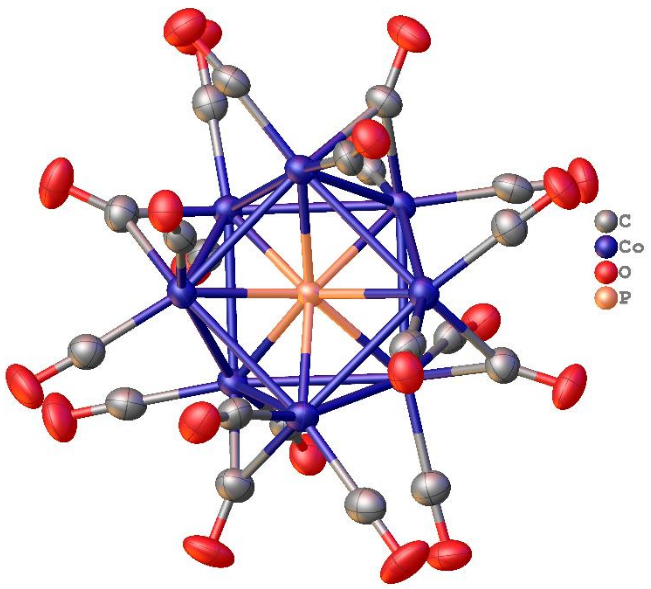

Fig. S17. View of the asymmetric unit of **5**.

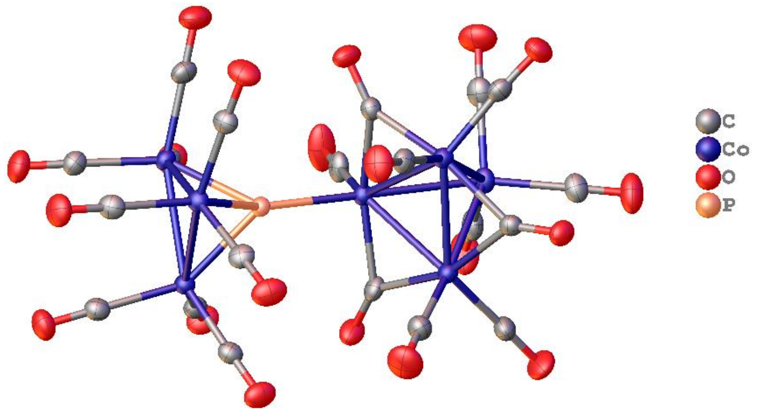

Fig. S18. View of the asymmetric unit of **6**.

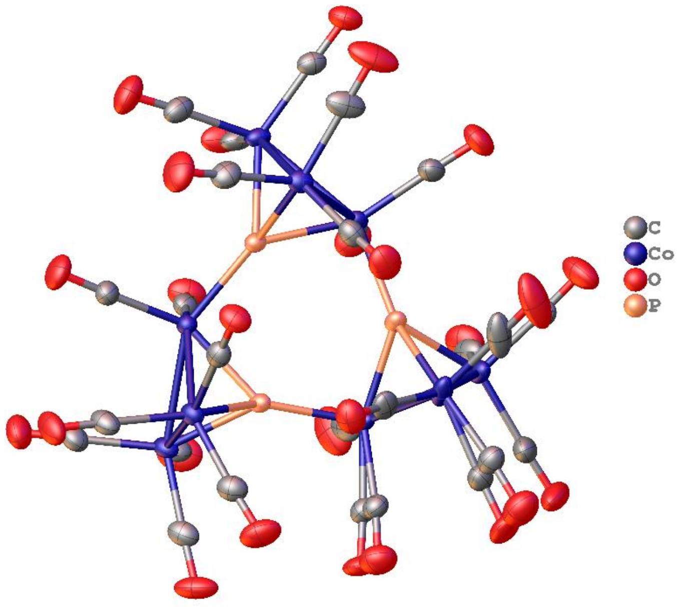

Fig. S19. View of the asymmetric unit of **7**.

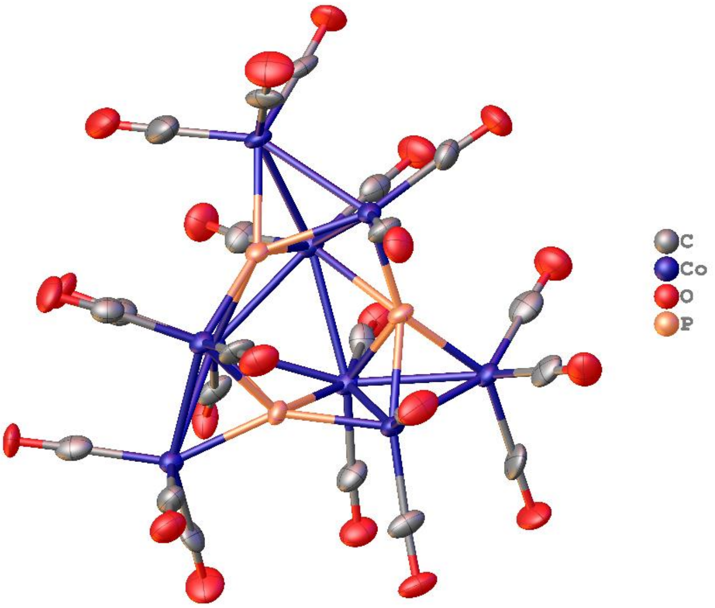

Fig. S20. View of the asymmetric unit of **8**.

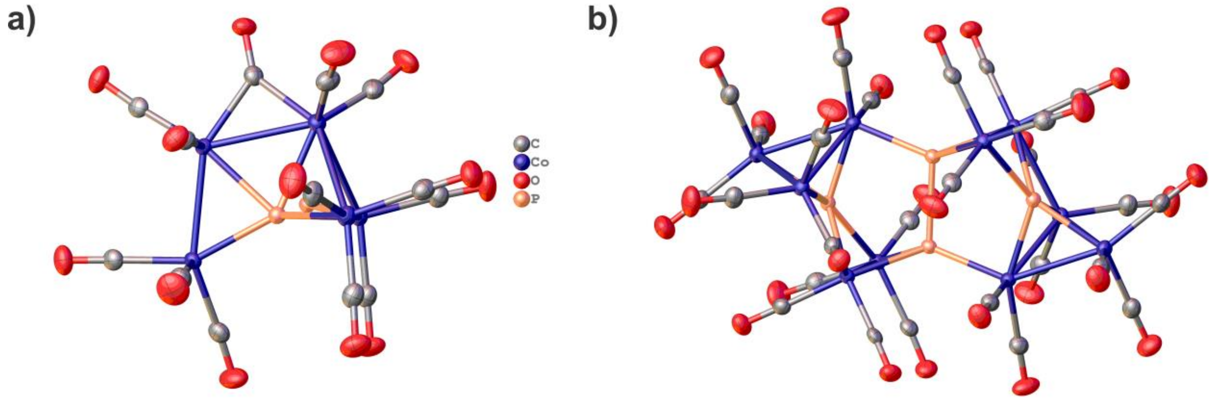

Fig. S21. a) View of the asymmetric unit of **9**; b) Molecular structure of compound **9** in the solid state.

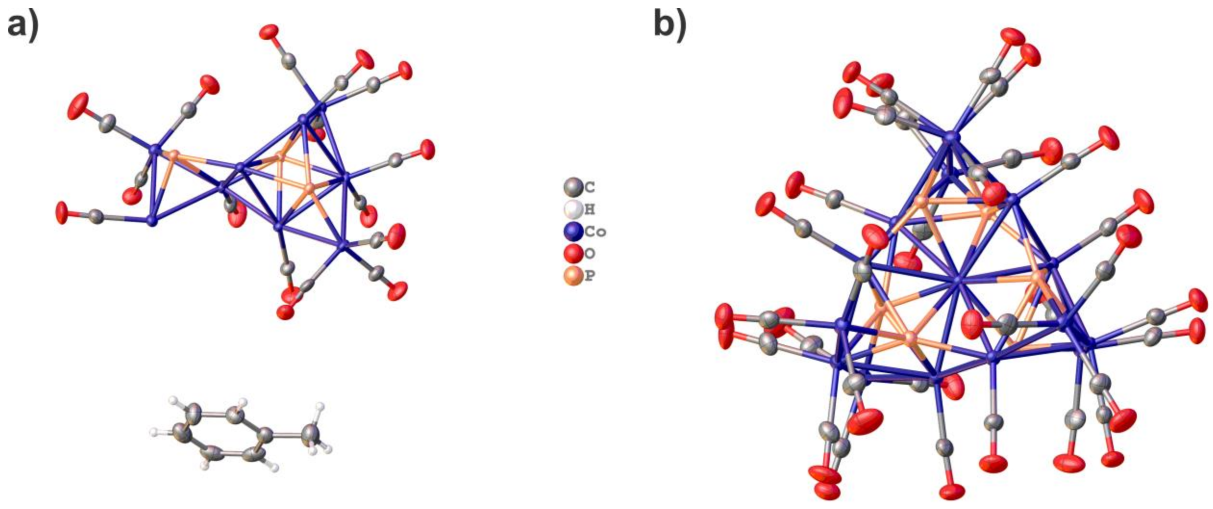

Fig. S22. a) View of the asymmetric unit of **10**; b) Molecular structure of compound **10** in the solid state.

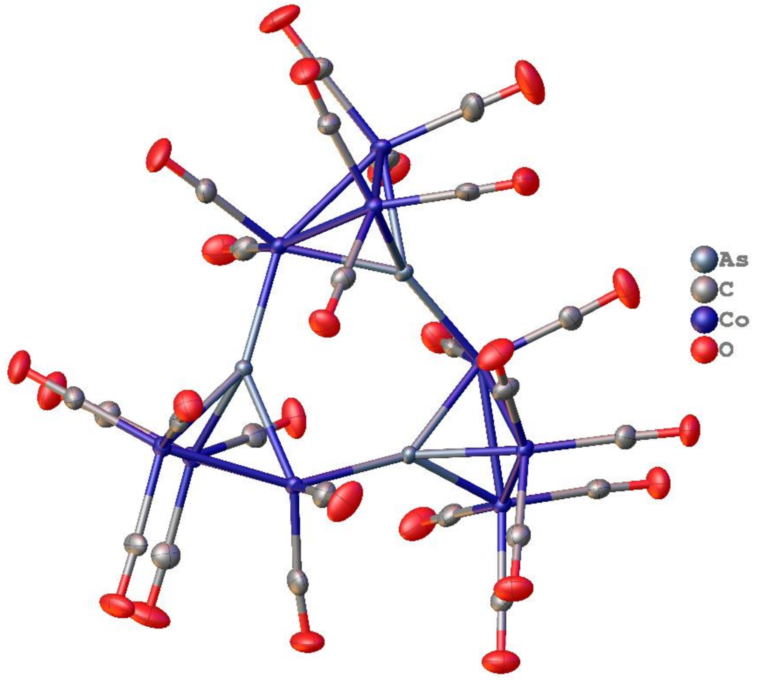

Fig. S23. View of the asymmetric unit of **11**.

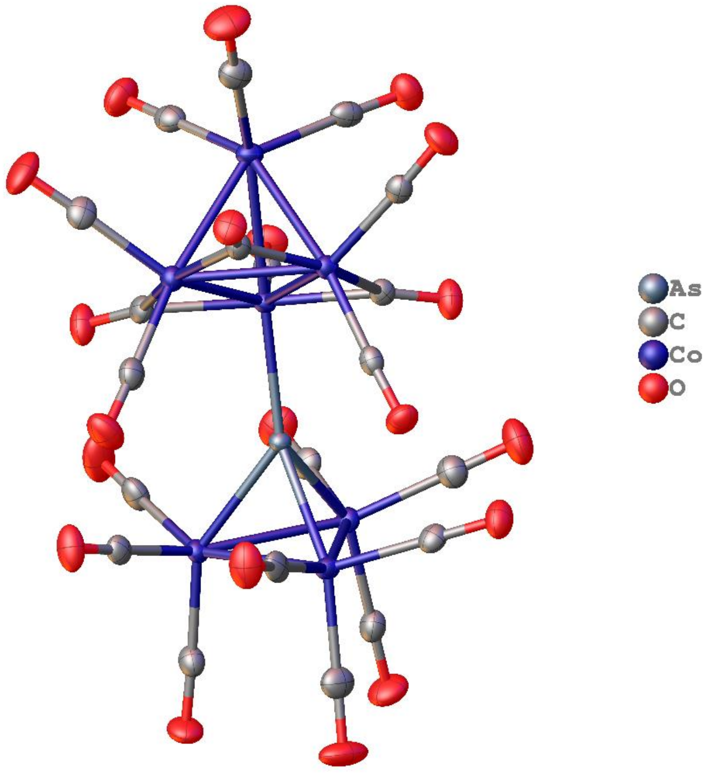

Fig. S24. View of the asymmetric unit of **12**.

References:

[1] a) R. A. Kendall, H. A. Früchtl, *Theor. Chem. Acc.* **1997**, 97, 158–163. b) F. Neese, *WIREs Comput. Mol. Sci.* **2012**, *2*, 73–78. c) F. Neese, *WIREs Comput. Mol. Sci.* **2018**, *8*, 1327.

[2] S. Grimme, A. Hansen, S. Ehlert, J.-M. Mewes, *J. Chem. Phys.* **2021**, *154*, 064103.

[3] H. Kruse, S. Grimme, *J. Chem. Phys.* **2012**, *136*, 154101.

[4] E. Caldeweyher, C. Bannwarth, S. Grimme, *J. Chem. Phys.* **2017**, *147*, 034112.

[5] Agilent Technologies Ltd, *CrysAlis PRO*, Yarnton, Oxfordshire, England, **2014**.

[6] O. V. Dolomanov, L. J. Bourhis, R. J. Gildea, J. A. K. Howard and H. Puschmann, *J. Appl. Crystallogr.* **2009**, *42*, 339–341.

[7] G. M. Sheldrick, *Acta Cryst. A* **2015**, *71*, 3–8.

[8] a) G. M. Sheldrick, *Acta Cryst. A* **2008**, *64*, 112–122; b) G. M. Sheldrick, *Acta Cryst. C* **2015**, *71*, 3–8.

[9] A. V.-Orosz, V. Galamb, G. Pályi, L. Markó, *J. Organomet. Chem.* **1976**, *107*, 235-240.

[10] H. Lang, G. Huttner, B. Sigwarth, I. Jibril, L. Zsolnai, O. Orama, *J. Organomet. Chem.* **1986**, *304*, 137-155.
